# Spatiotemporal Variability Analysis of PM_2.5_ and O_3_ Pollution Characteristics in the Fenwei Plain

**DOI:** 10.3390/toxics14050378

**Published:** 2026-04-28

**Authors:** Jingyue Xue, Yushuang Wang, Tingting Fan, Jian Peng

**Affiliations:** 1 School of Artificial Intelligence, China University of Geosciences, Beijing 100083, China; 1004235208@email.cugb.edu.cn (J.X.); 1004235209@email.cugb.edu.cn (T.F.); 1004235220@email.cugb.edu.cn (J.P.); 2 Hebei Key Laboratory of Geospatial Digital Twin and Collaborative Optimization, Beijing 100083, China

**Keywords:** Fenwei Plain, PM_2.5_, ozone (O_3_), spatiotemporal variability, compound pollution, GTWR model, synergistic control

## Abstract

The Fenwei Plain (FWP) is a typical basin-type region in northern China characterized by complex PM_2.5_ and O_3_ composite pollution. Based on hourly monitoring data from 11 cities (2015–2024), this study integrated the Mann–Kendall test, standard deviation ellipse (SDE), spatio-temporal cross-correlation function (STCCF), and spatio-temporal geographically weighted regression (GTWR) to systematically analyze decadal pollution patterns and coupling mechanisms. Results revealed a profound transition from particulate-dominated to photochemical-regime pollution: PM_2.5_ concentrations decreased significantly by 32%, whereas MDA8 O_3_ rose by 47%. Spring emerged as the critical compound pollution window, accounting for 66.7% of simultaneous exceedances. Spatially, both pollutants maintained a consistent Northeast–Southwest orientation, with PM_2.5_ hotspots concentrated along the Weihe River Valley. Cluster analysis categorized the 11 cities into O_3_-dominant, compound-high-risk, and PM_2.5_-dominant clusters. Furthermore, a dominant positive synergy was observed on an annual scale, although a localized “see-saw” effect occurred at a 150–200 km distance with a 3-year lag. The GTWR model demonstrated high robustness (R^2^: 0.75–0.86), underscoring the influence of localized driving forces. These findings provide a scientific basis for synergistic governance and precision air quality management in northern basin-type regions.

## 1. Introduction

Atmospheric pollution remains a paramount challenge for ecological civilization and public health. Fine particulate matter (PM_2.5_) and ozone (O_3_) have emerged as the primary pollutants affecting air quality, with their synergistic pollution trends attracting widespread academic attention [1,2,3]. PM_2.5_ poses severe risks to the respiratory and cardiovascular systems and reduces visibility, while O_3_, a secondary photochemical pollutant, damages ecosystems and human health through complex interactions with precursors under intense solar radiation [4,5]. The Fenwei Plain (FWP), a vital energy hub in the middle reaches of the Yellow River, is a typical basin-type geographic unit. Its unique “mountain-enclosed” structure, formed by the Lyuliang and Qinling mountains, frequently induces stagnant meteorological conditions that hinder pollutant dispersion [6,7]. Despite significant progress under the “Blue Sky Defense War,” which has successfully curtailed PM_2.5_ levels, O_3_ concentrations in the FWP have shown a continuous upward trend [8,9]. This transition toward a “PM-improvement, O_3_-rebound” phase necessitates a shift toward multi-pollutant synergistic control strategies [10].

Extensive research has explored the mechanisms and spatiotemporal patterns of these pollutants using increasingly sophisticated technical frameworks [11,12]. Methodologically, the field has evolved from simple spatial interpolation to advanced geostatistical and machine learning models capable of handling spatiotemporal non-stationarity. Globally, the non-linear regulation of precursors on O_3_ formation has been validated through box models and chemical transport models. To address spatial heterogeneity, Spatio-Temporal Geographically Weighted Regression (GTWR) has been widely adopted to confirm the significant local effects of topography and meteorology on PM_2.5_, outperforming traditional Ordinary Least Squares (OLS) and GWR models by incorporating the temporal dimension [13,14,15,16]. Furthermore, the application of Spatio-Temporal Cross-Correlation Functions (STCCF) has revealed critical time-lagged coupling between O_3_ and PM_2.5_ in urban clusters, providing a scientific basis for regional joint prevention and control [17].

In the Chinese context, research has primarily focused on the Beijing-Tianjin-Hebei (BTH) region and the Yangtze River Delta (YRD) [18,19,20,21]. Scholars utilized source apportionment and HYSPLIT models to delineate the seasonal peaks of PM_2.5_ driven by winter heating and long-range transport [22,23,24]. For O_3_, studies have highlighted the “south-high, north-low” spatial gradient across China, identifying temperature and solar radiation as the dominant meteorological drivers. Regarding the FWP, existing studies have identified the Weihe River Valley as a persistent PM_2.5_ hotspot and noted the diverging trends between particulate and photochemical pollution [25,26].

Despite these advancements, current research on the FWP predominantly focuses on short-term periods (typically under five years), failing to reflect long-term evolutionary trends. There remains a lack of quantitative insight into the multi-scale coupling mechanisms of PM_2.5_ and O_3_, and the integrated effects of basin topography, complex meteorology, and emission intensity are insufficiently quantified. To address these gaps, this study offers three primary innovations. First, by utilizing a comprehensive 10-year dataset (2015–2024), we systematically analyze the complete transition of the FWP’s pollution structure. Second, we integrate advanced models, including Standard Deviation Ellipse (SDE), STCCF, and GTWR, to reveal refined coupling mechanisms across specific spatial ranges (150–200 km) and temporal lags. Third, we identify the critical spring window for composite pollution and classify city-level pollution clusters using K-means algorithms. These findings provide a new scientific paradigm for atmospheric governance in northern basin-type regions and support the high-quality development of the Yellow River basin.

## 2. Materials and Methods

### 2.1. Study Area

The Fenwei Plain (FWP) (33°33′–38°42′ N, 106°20′–114°07′ E) is a representative basin-type pollution hotspot in northern China, encompassing 11 cities across three provinces: Lvliang (LL), Jinzhong (JZ), Linfen (LF), and Yuncheng (YC) in Shanxi; Xi’an (XA), Tongchuan (TC), Baoji (BJ), Xianyang (XY), and Weinan (WN) in Shaanxi; and Sanmenxia (SMX) and Luoyang (LY) in Henan. Topographically, the FWP is a flat interior basin enclosed by the Lvliang Mountains and the northern foothills of the Qinling Mountains [27]. This “mountain-enclosed” structure frequently induces stagnant meteorological conditions, such as thick inversion layers and low mixed-layer heights, which facilitate the localized accumulation of pollutants. As a major energy-industrial hub in the middle reaches of the Yellow River, the convergence of high emission intensity, complex terrain, and unfavorable meteorology makes the FWP a priority region for synergistic PM_2.5_ and O_3_ control in China [28,29].

### 2.2. Research Data

#### 2.2.1. Data Source

Atmospheric pollutant data, including concentrations of PM_2.5_ and MDA8 O_3_ (maximum daily 8 h average O_3_) from 2015 to 2024 for the 11 studied cities, were obtained from the China National Environmental Monitoring Centre (CNEMC, http://www.cnemc.cn/). Multiple monitoring stations are established for each pollutant, with data recorded at an hourly frequency.

Administrative boundary data for the study area were sourced from the National Geomatics Center of China (NGCC, http://www.ngcc.cn/). Using city-level administrative boundaries as vector data, raster data for the study area were extracted via mask operations in ArcGIS Pro 3.0. Subsequently, mean PM_2.5_ and O_3_ concentrations were statistically calculated across multiple timescales—including annual, seasonal, monthly, and daily intervals—to construct a comprehensive spatiotemporal sequence of pollutant concentrations for the Fenwei Plain from 2015 to 2024.

**Figure 1:**
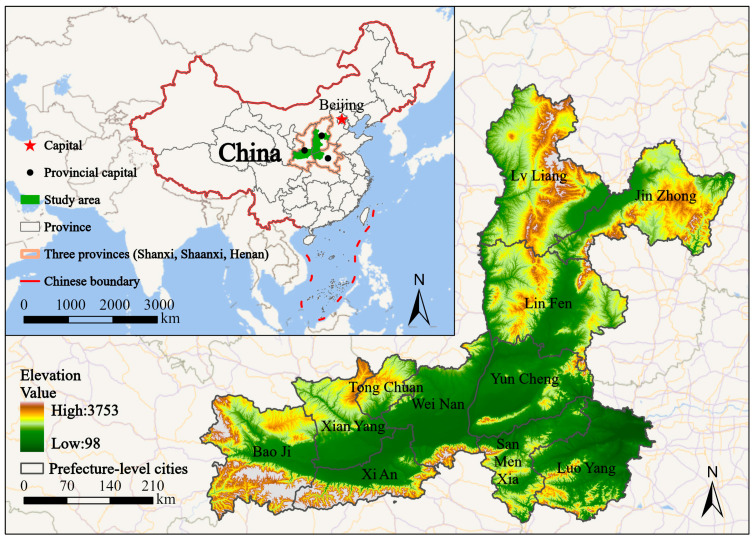
Overview map of the study area.

Digital Elevation Model (DEM) data used for the GTWR analysis were retrieved via the Open-Meteo Elevation API, which utilizes the CGIAR/SRTM 90 m (Version 4) dataset. This ensured that topographic parameters were extracted using the same coordinate-based sampling strategy and bilinear interpolation as the meteorological data, thereby reconciling the spatial resolution discrepancies at the station scale. Separately, the Google Earth Engine (GEE) platform was employed to process the same SRTM 90 m dataset specifically for generating high-resolution spatial visualizations, such as the study area overview map (Figure 1). Meteorological data were primarily sourced from the Open-Meteo open-source database (https://open-meteo.com/en/docs (accessed on 20 January 2026)), supplemented by data from the National Meteorological Science Data Center (https://data.cma.cn/). The acquired meteorological parameters include Mean Temperature (MT), Relative Humidity (RH), Mean Wind Speed (MW), Sunlight Duration (SD) and Precipitation (PRE). For occasional missing meteorological observations, linear interpolation was applied to ensure the continuity of the time series and the accuracy of the analysis.

#### 2.2.2. Data Processing

Based on the hourly PM_2.5_ and O_3_ monitoring data from 11 prefecture-level cities in the Fenwei Plain (2015–2024), data screening was initially performed according to the study’s spatial and temporal scope. First, invalid records and extreme outliers were excluded through concentration threshold verification. Subsequently, a stratified strategy was employed to handle missing values: for individual stations with a missing rate below 5%, linear interpolation was applied for gap-filling; conversely, stations with a missing rate exceeding 5% were removed entirely to ensure time-series continuity and reliability. Finally, derived indicators such as the daily maximum 8 h average O_3_ concentration (MDA8 O_3_) were calculated based on the cleaned raw data. Concentration mean datasets across multiple timescales—including annual, seasonal, monthly, and daily intervals—were then generated to provide high-quality standardized inputs for subsequent spatiotemporal pattern analysis and model development. Considering the aforementioned criteria and the spatial distribution of stations, a total of 55 air quality monitoring stations were ultimately selected for this research [30].

### 2.3. Methodology Overview

**Figure 2:**
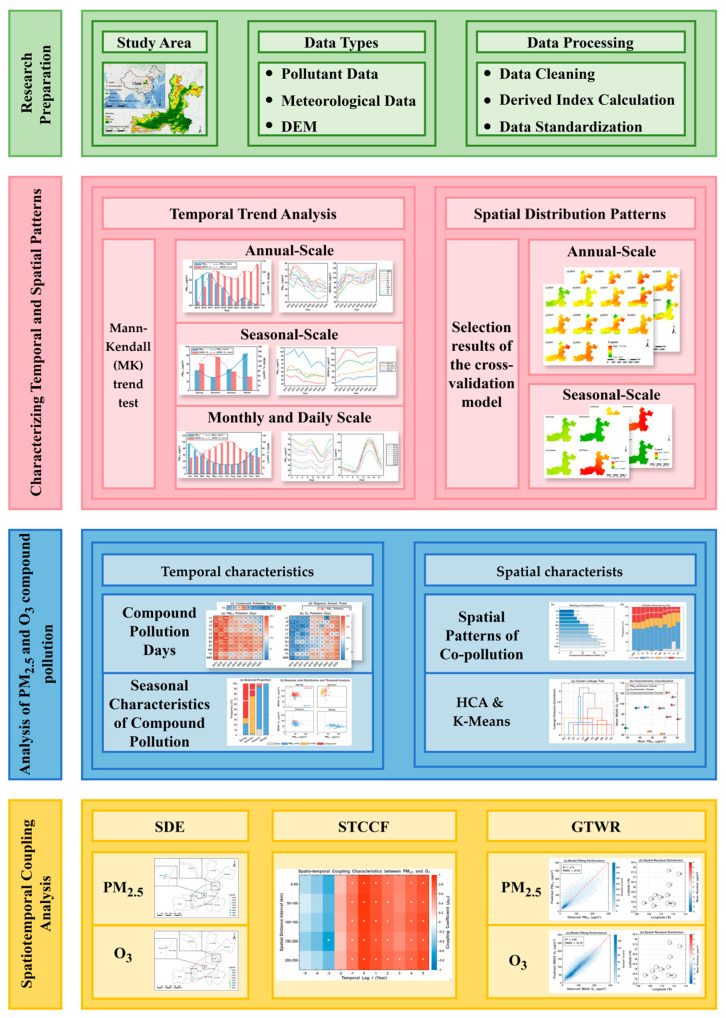
The methodology framework.

To systematically investigate the spatiotemporal dynamics and coupling relationships between PM_2.5_ and O_3_, a comprehensive research framework was designed as illustrated in Figure 2. The methodology is structured into four primary phases: (1) research preparation, including study area, data types and data processing; (2) characterizing temporal and spatial patterns of the pollutants; (3) analysis of PM_2.5_ and O_3_ compound pollution characteristics; and (4) spatiotemporal coupling analysis using advanced geostatistical and correlation models.

### 2.4. Research Methods

#### 2.4.1. Mann–Kendall (MK) Trend Test

The non-parametric Mann–Kendall (MK) test was employed to detect monotonic trends in PM_2.5_ and O_3_ time series. The MK test is highly robust against non-normal distributions and outliers, which are common in environmental data. The test statistic *S* is calculated as follows:

(1) $$S=\sum_{i=1}^{n-1}\sum_{j=i+1}^{n}sgn(x_{j}-x_{i})$$

where $x_{i}$ and $x_{j}$ are the sequential data values, *n* is the length of the dataset, and sgn(θ) is the sign function. For *n* > 10, the standardized statistic $Z_{MK}$ follows a standard normal distribution, computed as follows:

(2) $$Z_{MK}=\left\{\begin{array}{ll} \frac{S-1}{\sqrt{Var(S)}}, & if\;S>0\\ 0, & if\;S=0\\ \frac{S+1}{\sqrt{Var(S)}}, & if\;S<0 \end{array}\right.$$

The *p*-value derived from $Z_{MK}$ was used to evaluate the statistical significance of the trend. In this study, significance levels of α=0.05 and α=0.01 were adopted. A positive $Z_{MK}$ indicates an upward trend, while a negative value represents a downward trend. Specifically, $|Z_{MK}|>1.96$ and $|Z_{MK}|>2.58$ represent significant (*p* < 0.05) and highly significant (*p* < 0.01) trends, respectively.

#### 2.4.2. Spatial Interpolation and Model Validation

(1) Ordinary Kriging (OK)

Ordinary Kriging (OK) is a widely utilized geostatistical interpolation method for spatial estimation [31]. Its fundamental premise is the assumption of second-order stationarity, which posits that the attribute mean is a constant unknown across the study area, and the spatial autocorrelation depends solely on the distance between any two locations rather than their absolute positions [32].

The OK method characterizes the spatial structure of the data using a semivariogram. Under the constraint of an unbiasedness condition (where the sum of weights equals 1), the method minimizes the estimation variance to achieve the Best Linear Unbiased Estimation (BLUE). For an unsampled location $x_{0}$, the attribute value $\hat{Z}(x_{0})$ is estimated as a linear combination of the observed values at neighboring sample points:

(3) $$\hat{Z}(x_{0})=\sum_{i=1}^{n}\lambda_{i}Z(x_{i}),\;subject\;to\sum_{i=1}^{n}\lambda_{i}=1$$

where $Z(x_{i})$ represents the observed value at the i-th sample point, *n* is the number of sample points involved in the interpolation, and $\lambda_{i}$ denotes the weight assigned to each point. The weights $\lambda_{i}$ are determined by solving the following Kriging system of equations:

(4) $$\left\{\begin{array}{l} \sum_{j=1}^{n}\lambda_{j}C(x_{i},x_{j})+\mu =C(x_{i},x_{0})\;(i=1,2,\ldots ,n)\\ \sum_{i=1}^{n}\lambda_{i}=1 \end{array}\right.$$

In this system, $C\left(x_{i},x_{j}\right)$ is the covariance between sample points $x_{i}$ and $x_{j}$, and μ represents the Lagrange multiplier used to minimize the error variance.

To formally define the spatial structure, $C\left(h\right)$ is the spatial autocovariance function for a lag distance h, which characterizes the statistical correlation between two spatial variables. It is defined as follows:

(5) $$C\left(h\right)=E\left[Z\left(x\right)\cdot Z\left(x+h\right)\right]-m^{2}$$

where E[·] denotes the mathematical expectation and m is the stationary mean.

The relationship between the covariance $C\left(h\right)$ and the semivariogram γ(h) is defined as follows:

(6) C(h)=C(0)−γ(h)

where $C\left(0\right)$ is the variance of the data, which corresponds to the Sill in the variogram model. The semivariogram γ(h) is determined by fitting a theoretical model (e.g., Gaussian, Spherical, or Exponential models) to the experimental semivariogram derived from the sample data:

(7) $$\gamma (h)=\frac{1}{2N(h)}\sum_{i=1}^{N(h)}[Z(x_{i})-Z(x_{i}+h){]}^{2}$$

where $N\left(h\right)$ is the number of pairs of sample points separated by distance h.

(2) Model Performance Evaluation and Cross-validation

To ensure the predictive accuracy and reliability of the Ordinary Kriging (OK) models, a Leave-One-Out Cross-Validation (LOOCV) procedure was implemented [33,34,35]. This method involves sequentially removing each monitoring station and using the remaining *n* − 1 stations to predict the concentration at the excluded location. The interpolation performance was quantitatively assessed across multiple dimensions—unbiasedness, stability, and predictive capability—using five statistical diagnostic metrics: Mean Error (ME), Root Mean Square Error (RMSE), Average Standard Error (ASE), Mean Standardized Error (MSE), and Root Mean Square Standardized Error (RMSSE). The mathematical definitions and optimal value thresholds for these parameters are summarized in Table 1. Specifically, an ideal interpolation result is characterized by an ME and MSE close to 0, a minimized RMSE, and an RMSSE approaching 1. These metrics serve as the scientific basis for selecting the optimal theoretical semivariogram model for PM_2.5_ and O_3_ mapping.

#### 2.4.3. Methods of PM_2.5_ and O_3_ Compound Pollution Analysis

(1) Definition of Compound Pollution Days

To systematically quantify the dynamic coupling characteristics of PM_2.5_ and O_3_ at multiple timescales, this study constructed a daily air quality classification system for the Fenwei Plain from 2015 to 2024. This system integrates guidelines from the World Health Organization (WHO) and current Chinese ambient air quality standards. Specifically, a Daily mean PM_2.5_ concentration $>75\;\mu g/m^{3}$ and a Maximum Daily 8 h Average (MDA8) O_3_ concentration $>160\;\mu g/m^{3}$ were defined as the exceedance thresholds for each pollutant, respectively.

Based on these dual thresholds, data records were categorized into four distinct types:

1. Compound Pollution Days: Dates when both PM_2.5_ and O_3_ concentrations simultaneously exceeded their respective limits, reflecting periods of intensified risk due to dual-pollutant superposition.
2. PM_2.5_-only Pollution Days: Dates when only PM_2.5_ concentrations exceeded the threshold.
3. O_3_-only Pollution Days: Dates when only O_3_ concentrations exceeded the threshold.
4. Clean Days: Dates when concentrations of both pollutants remained below their respective limits.

This classification system anchors the regulatory targets with national concentration limit values and systematically delineates the synergistic characteristics of PM_2.5_ and O_3_. Furthermore, it establishes a high-quality standardized foundation for the subsequent spatial analysis of pollution patterns and model development in the study area.

(2) Integrated Spatial Clustering Strategy (HCA & K-Means)

To objectively identify city clusters with distinct compound pollution patterns, this study employed an integrated spatial clustering strategy, combining Agglomerative Hierarchical Clustering (HCA) and K-Means clustering algorithm [36,37]. The input features for clustering comprised the multi-year (2015–2024) mean concentrations of PM_2.5_ and MDA8 O_3_, and the compound exceedance ratio for each of the 11 monitored cities. All input features were normalized (z-score standardization) prior to analysis.

First, HCA was implemented to discover the natural hierarchical structure of the city dataset. This unsupervised method iteratively merges the closest pairs of clusters based on their spatial distance. A linkage tree (Dendrogram) was generated to visualize these relationships, where the X-axis represents individual cities and the Y-axis denotes the Euclidean linkage distance (indicating the degree of dissimilarity). The branch heights correspond directly to the distance at which clusters are merged, with higher branches signifying greater differences in compound pollution characteristics between the merged clusters. Crucially, the distinct branching structure of the dendrogram provided a clear, intuitive, and objective basis for determining the optimal number of clusters (K) for the subsequent analysis, rather than using arbitrary selection.

Subsequently, based on the natural cluster divisions identified by HCA, the K-Means algorithm was applied to achieve the final spatial classification. To quantitatively validate the optimal number of clusters (K), we employed the Elbow Method and Silhouette Analysis. The K-Means algorithm aimed to minimize the Within-Cluster Sum of Squares (WCSS). The objective function J is defined as follows:

(8) $$J=\sum_{k=1}^{K}\sum_{i\in C_{k}}\|x\prime_{i}-\mu_{k}{\|}^{2}$$

where $C_{k}$ is the set of cities in the k-th cluster, $x\prime_{i}$ is the normalized feature vector of the i-th city, and $\mu_{k}$ is the centroid (mean vector) of the k-th cluster.

The Elbow Method was used to observe the rate of decrease in WCSS as K increased. A distinct “elbow” point was identified at K=3, where the WCSS was 6.20, and the addition of more clusters yielded diminishing returns in further reducing the internal variance. Furthermore, the Silhouette Coefficient (S), which measures the cohesion of clusters and the separation between them, was calculated to assess the clustering quality. The average silhouette value reached its maximum at K=3 (approx.0.394). Considering the spatial continuity and regional transport of atmospheric pollutants, this value confirms that the three-cluster partition provided a robust and well-separated cluster structure for the Fenwei Plain.

The algorithm optimizes the final partitions through iterative two-step refinement:

1. Assignment Step: Each city i is assigned to the cluster whose centroid is closest to its standardized vector, according to the condition:
  (9) $$C_{k}=\{i:\|x\prime_{i}-\mu_{k}\|\leq \|x\prime_{i}-\mu_{j}\|,\forall j\neq k\}$$
2. Update Step: The centroids of each of the K clusters are recalculated based on their assigned members.

Through this integrated approach, the cities across the Fenwei Plain were robustly partitioned into three distinct groups: PM_2.5_-dominant, O_3_-dominant, and Compound-dominant pollution hotspots. This objective spatial grouping directly informed the development of differentiated, zonally targeted dual-control strategies tailored to each cluster’s unique pollution drivers.

#### 2.4.4. Methods of Spatio-Temporal Interaction and Coupling Analysis

(1) Standard Deviation Ellipse (SDE)

The Standard Deviation Ellipse (SDE) was employed to characterize the spatio-temporal evolution of PM_2.5_ and O_3_ distributions [38,39,40]. By quantifying parameters such as the mean center, major/minor axes, and rotation angle, the SDE effectively captures the spatial concentration, orientation, and dispersion trends of pollutants. The weighted mean center (*X*, *Y*) is defined as follows:

(10) $$X=\frac{\sum_{i=1}^{n}w_{i}x_{i}}{\sum_{i=1}^{n}w_{i}},Y=\frac{\sum_{i=1}^{n}w_{i}y_{i}}{\sum_{i=1}^{n}w_{i}}$$

where $\left(x_{i},y_{i}\right)$ are the spatial coordinates of city i, and $w_{i}$ represents the pollutant concentration as the spatial weight. The lengths of the major and minor axes are derived from the eigenvalues ($\lambda_{1},\lambda_{2}$) of the weighted covariance matrix, representing the primary and secondary directions of pollution spread, respectively. The rotation angle θ indicates the orientation of the pollution corridor relative to true north.

(2) Spatio-Temporal Cross-Correlation Function (STCCF)

To investigate the multi-year synergistic evolution and potential “memory effects” between PM_2.5_ and O_3_, the Spatio-Temporal Cross-Correlation Function (STCCF) was utilized. Unlike traditional correlation analysis, STCCF quantifies the interaction intensity between these two pollutants across various temporal lags (τ) and spatial distance intervals (*d*).

For the normalized annual sequences of PM_2.5_ (*X*) and O_3_ (*Y*), the coupling coefficient STCCF(τ,d) is defined as follows:

(11) $$STCCF(\tau ,d)=\frac{1}{N_{d}}\sum_{(i,j):D_{ij}\in d}c\;or(X_{i,t}^{*},Y_{j,t+\tau }^{*})$$

where

1. Temporal Lag (τ): Set from −5 to 5 years. A positive τ indicates the potential impact of PM_2.5_ on subsequent O_3_ levels, while a negative τ suggests the reverse.
2. Spatial Distance (*d*): To capture the regional transport scale within the Fenwei Plain, the spatial distance was categorized into five 50 km intervals: 0–50 km, 50–100 km, 100–150 km, 150–200 km, and 200–250 km.
3. $N_{d}$: Represents the number of city pairs within each spatial bin d.
4. $cor(X_{i,t}^{*},Y_{j,t+\tau }^{*})$ represents the Pearson correlation coefficient between the normalized sequences of the two pollutants.
5. The asterisk (*) denotes that the raw data sequences have been normalized (via zero-mean and unit-variance scaling) prior to the calculation, ensuring the variables are dimensionless and comparable.

To ensure the reliability of the observed coupling patterns, a Permutation Test (1000 iterations) was performed. Significant spatio-temporal correlations (p<0.05) are identified by white asterisks (*) in the resulting heatmap. This method effectively isolates whether the observed PM_2.5_-O_3_ coupling is a localized phenomenon or a long-range, delayed regional interaction.

(3) Geographically and Temporally Weighted Regression (GTWR)

The Geographically and Temporally Weighted Regression (GTWR) model was implemented to capture the spatio-temporal non-stationarity of the drivers influencing PM_2.5_ and O_3_ concentrations [41,42]. By incorporating a temporal dimension into the traditional GWR, GTWR generates local rather than global regression coefficients:

(12) $$y_{i}=\beta_{0}(u_{i},v_{i},t_{i})+\sum_{k=1}^{p}\beta_{k}(u_{i},v_{i},t_{i})x_{ik}+\varepsilon_{i}$$

where $\left(u_{i},v_{i},t_{i}\right)$ represents the spatio-temporal coordinates of the i-th observation; p denotes the total number of explanatory variables, and k represents the index of each independent variable. The core of GTWR is the construction of a spatio-temporal weight matrix $w_{ij}$ using a Gaussian kernel:

(13) $$w_{ij}=\exp\left[-\frac{1}{2}{\left(\frac{d_{s,ij}}{b_{s}}\right)}^{2}-\frac{1}{2}{\left(\frac{d_{t,ij}}{b_{t}}\right)}^{2}\right]$$

where $d_{s,ij}$ and $d_{t,ij}$ are the spatial and temporal distances, respectively. The spatial bandwidth ($b_{s}$) and temporal bandwidth ($b_{t}$) were optimized to 0.8 and 1.0, respectively, ensuring that the model captures localized variations while maintaining statistical robustness.

## 3. Results

### 3.1. Temporal Variation Characteristics

#### 3.1.1. Results of Time Series Trend Analysis

Between 2015 and 2024, air pollution patterns in the Fenwei Plain underwent a significant shift [43]. MK test results, which are presented in Table 2, indicated a consistent and significant downward trend in PM_2.5_ concentrations across the region and in individual cities, with a regional Z=−3.04 (p<0.01). This reduction was most pronounced in cities such as SMX, YC, and TC, validating the efficacy of emission control policies like the “Blue Sky Defense War.”

Conversely, MDA8 O_3_ concentrations exhibited an overall increasing trend (Z=1.79). Although this did not reach the 95% significance level, it approached the threshold. At the city level, O_3_ levels in JZ and XA rose significantly, while SMX and XY showed near-significant upward trends. In contrast, only TC recorded a non-significant decrease, potentially due to industrial restructuring and intensive precursor emission reductions. Overall, the region has transitioned into a phase characterized by “PM_2.5_ mitigation and O_3_ rebound.” Future strategies should therefore shift from PM_2.5_-centric controls to synergistic management of both PM_2.5_ and O_3_, specifically targeting precise NOx and VOCs reductions [44,45,46].

#### 3.1.2. Annual-Scale Variation Trends

**Figure 3:**
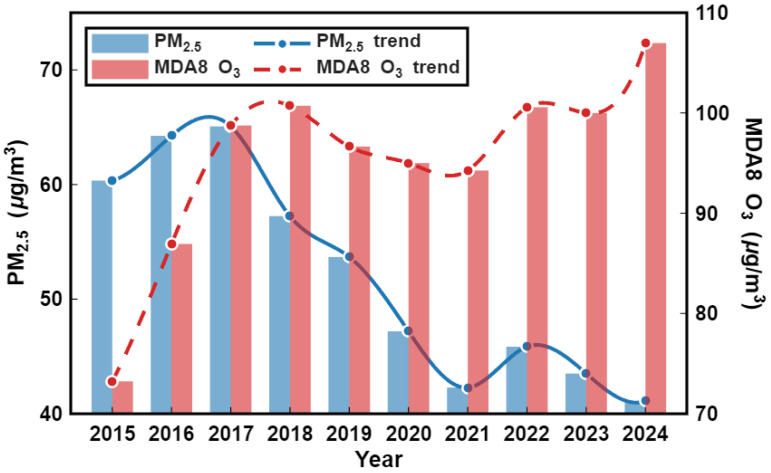
Temporal trends of annual average PM_2.5_ and MDA8 O_3_ concentrations in the Fenwei Plain from 2015 to 2024.

Figure 3 illustrates the interannual variation characteristics of two typical atmospheric pollutants in the Fenwei Plain from 2015 to 2024, clearly revealing a transition in regional air pollution patterns. The PM_2.5_ concentration exhibited a significant declining trend, fluctuating from approximately 60 $\mu {g/m}^{3}$ in 2015 to 41 $\mu {g/m}^{3}$ in 2024, representing a reduction of about 32%. This decline reflects the effective control of fine particulate matter resulting from a series of mitigation measures, such as industrial restructuring and energy structure optimization in recent years. In sharp contrast, the MDA8 O_3_ concentration showed a fluctuating upward trajectory, rising from approximately 75 $\mu {g/m}^{3}$ in 2015 to 110 $\mu {g/m}^{3}$ in 2024. This 47% increase indicates that ozone has emerged as the primary challenge for current regional air quality management.

**Figure 4:**
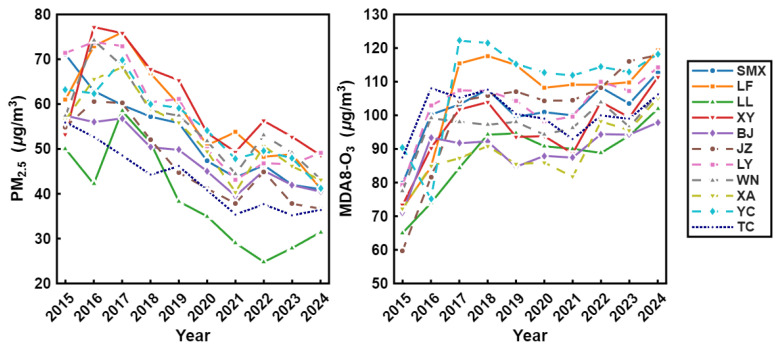
Temporal trends of annual average PM_2.5_ (**left**) and MDA8 O_3_ (**right**) concentrations in various cities of the Fenwei Plain from 2015 to 2024.

Figure 4 illustrates the temporal evolution of annual mean PM_2.5_ and MDA8 O_3_ concentrations across typical cities in the Fenwei Plain from 2015 to 2024. Contrary to the rising trajectory of MDA8 O_3_, PM_2.5_ concentrations in all cities exhibited a significant declining trend, highlighting the remarkable efficacy of regional emission reduction measures. Specifically, LL and TC recorded the most substantial decreases; in LL, the concentration dropped from approximately ~50 $\mu {g/m}^{3}$ in 2015 to about ~30 $\mu {g/m}^{3}$ in 2024, representing a reduction of over 40%. Similarly, concentrations in SMX and YC decreased from over 70 $\mu {g/m}^{3}$ initially to below 50 $\mu {g/m}^{3}$ by 2024. Despite minor short-term fluctuations in some cities, the consistent downward trend indicates that PM_2.5_ pollution in the Fenwei Plain has been effectively curtailed.

Overall, MDA8 O_3_ concentrations in all cities showed a fluctuating upward trend, revealing that ozone has emerged as a burgeoning challenge for regional air quality. Notably, YC and LF experienced the most significant increases, with the peak concentration in YC exceeding 120 $\mu {g/m}^{3}$ and LF approaching 120 $\mu {g/m}^{3}$ in 2024. Although LL maintained relatively lower overall levels, it also exhibited a steady rise from approximately 65 $\mu {g/m}^{3}$ in 2015 to 102 $\mu {g/m}^{3}$ in 2024. While the magnitude of change varied by city, the collective upward trajectory underscores the intensifying pressure of ozone pollution, which is consistent with the significant upward trends identified by the Mann–Kendall (MK) test.

#### 3.1.3. Seasonal-Scale Variation Results

**Figure 5:**
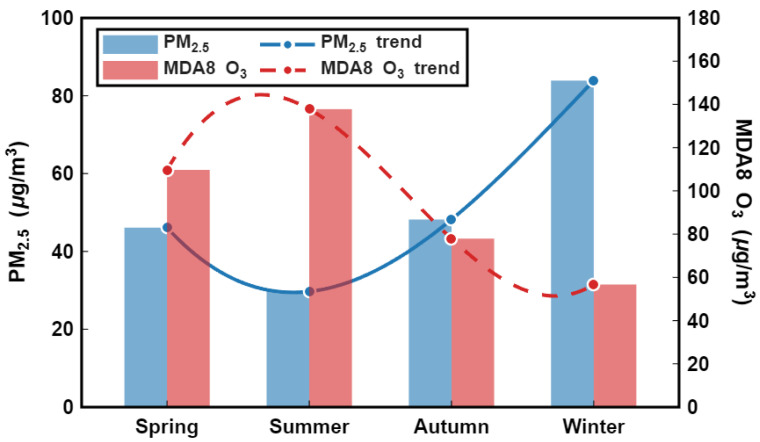
Seasonal variations of PM_2.5_ and MDA8 O_3_ concentrations in the Fenwei Plain.

Figure 5 clearly illustrates the seasonal distribution of the two primary atmospheric pollutants in the Fenwei Plain, revealing significant seasonal heterogeneity and transition patterns. MDA8 O_3_ concentrations peaked in summer ($\sim 140\;\mu {g/m}^{3}$), substantially exceeding other seasons due to intensive photochemical activity under high temperatures and strong solar radiation. Concentrations were secondary in spring ($\sim 110\;\mu {g/m}^{3}$) and declined markedly in autumn ($\sim 80\;\mu {g/m}^{3}$) and winter ($\sim 55\;\mu {g/m}^{3}$), reflecting the strong dependence of ozone formation on meteorological factors. In sharp contrast, ${PM}_{2.5}$ followed a typical “winter-high and summer-low” pattern: concentrations reached a maximum in winter ($\sim 85\;\mu {g/m}^{3}$), remained comparable in autumn ($\sim 50\;\mu {g/m}^{3}$) and spring ($\sim 45\;\mu {g/m}^{3}$), and dropped to a minimum in summer ($\sim 30\;\mu {g/m}^{3}$). This distribution pattern is attributed to enhanced emissions during the winter heating period, as well as the beneficial effects of precipitation scavenging and improved dispersion conditions in summer. These seasonal dynamics confirm that air pollution in the Fenwei Plain is dominated by ${PM}_{2.5}$ in winter and shifts to $O_{3}$ in summer, with both pollutants exerting synergistic effects during spring and autumn.

**Figure 6:**
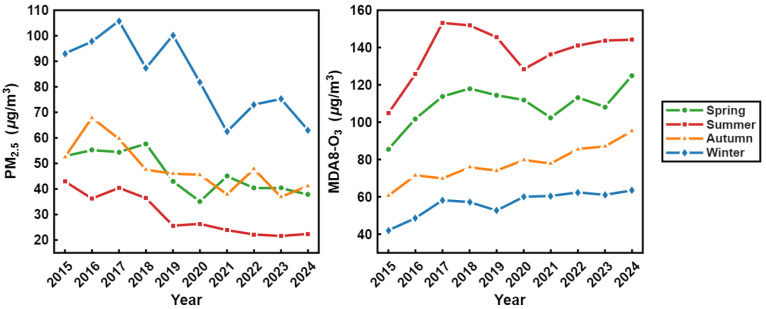
Interannual variation trends of seasonal mean PM_2.5_ (**left**) and MDA8 O_3_ (**right**) concentrations in the Fenwei Plain from 2015 to 2024.

From 2015 to 2024, the seasonal concentration trends of PM_2.5_ and MDA8 O_3_ in the Fenwei Plain clearly reveal distinct seasonal heterogeneity and shifting interannual patterns (Figure 6). PM_2.5_ concentrations consistently followed a typical pattern of peaking in winter and reaching minimums in summer. Furthermore, interannual winter concentrations exhibited a fluctuating downward trend, reflecting the superposition effect of emissions during the winter heating period and unfavorable dispersion conditions, while also verifying the effectiveness of regional emission reduction measures in curbing particulate pollution. In sharp contrast, MDA8 O_3_ concentrations displayed a seasonal distribution characterized by summer peaks and winter lows, with interannual summer concentrations showing a fluctuating upward trajectory. This demonstrates the strong dependence of ozone formation on meteorological conditions marked by high temperatures and intense solar radiation.

#### 3.1.4. Monthly and Daily Scale Variation Results

**Figure 7:**
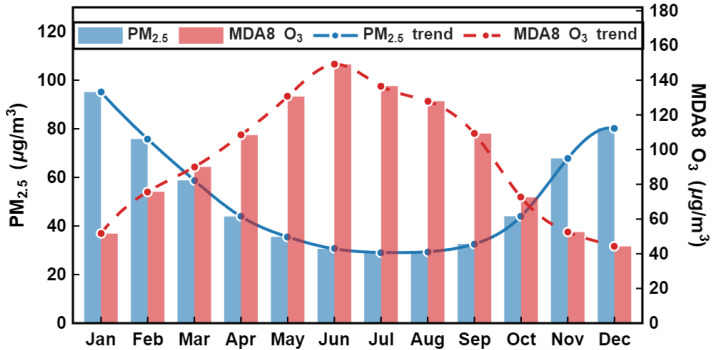
Monthly variations of PM_2.5_ and MDA8 O_3_ concentrations in the Fenwei Plain from 2015 to 2024.

Figure 7 illustrates the intra-annual variation in monthly mean PM_2.5_ and MDA8 O_3_ concentrations in the Fenwei Plain from 2015 to 2024. The left and right vertical axes represent the concentrations of PM_2.5_ and MDA8 O_3_ ($\mu {g/m}^{3}$), respectively.

As shown, ${PM}_{2.5}$ concentrations exhibited a pronounced “winter-high and summer-low” pattern. Annual peaks were recorded in January ($∼95\;\mu {g/m}^{3}$) and December ($∼80\;\mu {g/m}^{3}$), while concentrations declined significantly during the summer (June–August), reaching an annual minimum in July ($\sim 30\;\mu {g/m}^{3}$). This seasonal cycle is closely associated with enhanced emissions from winter heating and unfavorable atmospheric dispersion conditions, as well as the effective scavenging effect of increased precipitation during the summer months.

Conversely, MDA8 O_3_ concentrations exhibited a contrasting seasonal pattern, peaking during the summer months with values reaching approximately 140 $\mu {g/m}^{3}$. This summer maximum is primarily attributed to enhanced photochemical reactions driven by stronger solar radiation and higher temperatures, which accelerate the conversion of precursors into ground-level ozone. In contrast, the lower O_3_ levels observed in winter are linked to reduced photolysis rates and the titration effect of NO under stable atmospheric conditions.

**Figure 8:**
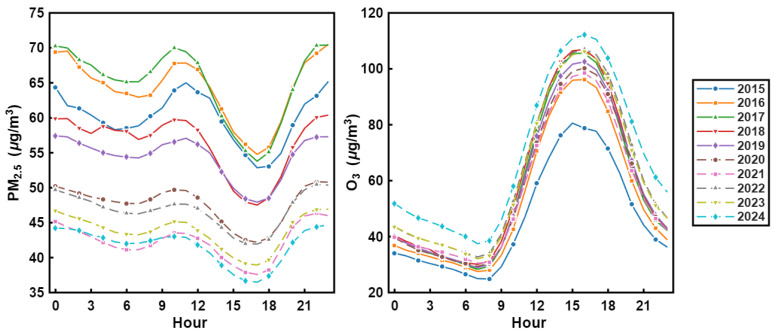
Diurnal variations of hourly mean PM_2.5_ (**left**) and O_3_ (**right**) concentrations in the Fenwei Plain from 2015 to 2024.

The left panel illustrates the diurnal variation characteristics of hourly mean ${PM}_{2.5}$ concentrations in the Fenwei Plain from 2015 to 2024 (Figure 8). Overall, PM_2.5_ exhibited a typical “bimodal” diurnal pattern. Two concentration peaks were observed during the morning (9:00–11:00) and evening (21:00–23:00), which are primarily attributed to increased traffic emissions during rush hours and the compression of the planetary boundary layer (PBL) height, leading to unfavorable dispersion conditions. Conversely, concentration valleys occurred in the afternoon (16:00–18:00) and predawn (5:00–7:00). The afternoon minimum is associated with a higher PBL and enhanced thermal turbulence favoring pollutant dilution, while the predawn low reflects the gradual dispersion of overnight accumulation.

The right panel presents the corresponding diurnal profiles for O_3_ during the same period, showing a prominent “unimodal” distribution. O_3_ concentrations remained low from night to early morning (0:00–7:00). With the intensification of solar radiation and rising temperatures, photochemical reactions accelerated, causing a rapid increase in O_3_ that peaked in the afternoon (15:00–17:00). Subsequently, concentrations declined in the evening as solar radiation weakened and the consumption of O_3_ through NOx titration increased.

### 3.2. Spatial Variation Characteristics

#### 3.2.1. Selection Results of the Cross-Validation Model

The interpolation accuracy of Ordinary Kriging (OK) highly depends on the structural characteristics of the semivariogram (the mathematical expression of spatial autocorrelation). Therefore, the core objective of cross-validation is not merely the optimization of all parameters, but rather the identification of the optimal semivariogram model that best fits the spatial distribution characteristics of PM_2.5_ and O_3_ concentrations in the study area, while simultaneously verifying the rationality of the model parameters.

In this study, four classic semivariogram models—circular, Gaussian, spherical, and exponential—were fitted based on the training dataset to determine key structural parameters, including the nugget (C_0_), range (a), and sill (C_0_ + C). A comprehensive evaluation was conducted using five geostatistical cross-validation metrics: Mean Error (ME), Root Mean Square Error (RMSE), Average Standard Error (ASE), Mean Standardized Error (MSE), and Root Mean Square Standardized Error (RMSSE). The optimal semivariogram model was selected based on the criteria that ME and MSE approach 0, RMSE is minimized, RMSSE approaches 1, and ASE is closest to RMSE. This selected model serves as the final theoretical framework for the spatial interpolation of PM_2.5_ and MDA8 O_3_ in the Fenwei Plain from 2015 to 2024.

As illustrated in Table 3, the interpolation accuracies of the four semivariogram models for PM_2.5_ and MDA8 O_3_ exhibit distinct variations. For PM_2.5_, the Exponential model achieved the lowest RMSE (10.547), which was notably superior to the Circular (10.869), Gaussian (10.858), and Spherical (10.860) models. While the RMSSE of the Circular model (1.00459) was the closest to the ideal value of 1, the Exponential model’s RMSSE (0.970326) also remained within an acceptable range with high reliability.

Regarding MDA8 O_3_, the Exponential model also demonstrated the best performance in terms of unbiasedness. Specifically, its RMSE (12.189) was among the lowest, while the ME (−0.024892) and MSE (0.001127) were both near zero, suggesting negligible systematic bias. Consequently, considering both predictive accuracy and unbiasedness, the Exponential model was selected as the optimal semivariogram for the spatial interpolation of PM_2.5_ and MDA8 O_3_. This model will be employed for the subsequent simulation of the spatial distribution of air pollutants across the Fenwei Plain from 2015 to 2024.

#### 3.2.2. Spatial Distribution at an Annual Scale

**Figure 9:**
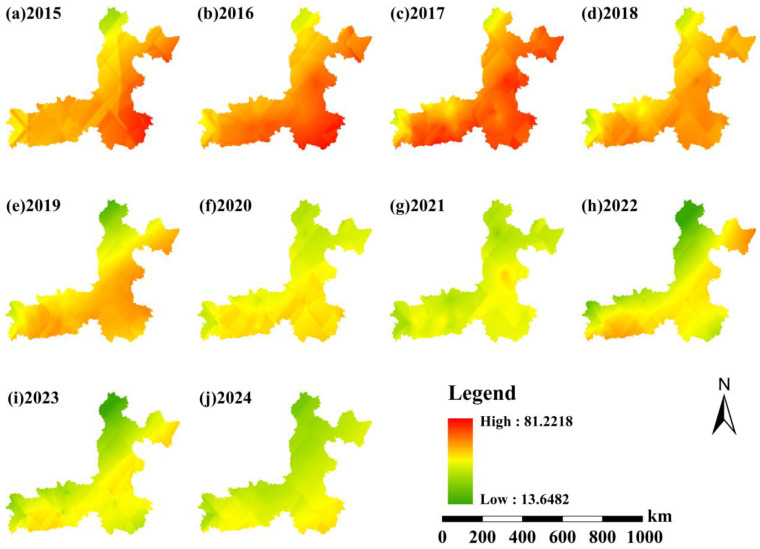
Spatial distribution of annual mean PM_2.5_ concentrations in the Fenwei Plain (2015–2024).

The spatial distribution of annual mean PM_2.5_ concentrations in the Fenwei Plain exhibits significant regional heterogeneity, with pollution intensity showing a distinct temporal gradient (Figure 9). As illustrated in the figure, high-concentration areas are primarily concentrated in the southern and western regions of the study area, encompassing cities such as BJ, XY, XA, and WN. These areas form a continuous pollution belt along the Weihe River Valley. This spatial clustering is closely associated with topographic blocking, intensive anthropogenic emissions (e.g., industrial clusters and vehicular traffic), and unfavorable meteorological conditions for pollutant dispersion.

From 2015 to 2024, PM_2.5_ concentrations across the entire plain demonstrated a significant downward trend. In 2015, large-scale areas exceeded the national annual air quality standards, with peak concentrations in core urban centers surpassing $80\;\mu g/m^{3}$. By 2023, the extent of high-pollution zones had contracted substantially, the overall concentration gradient had weakened, and air quality had improved markedly.

**Figure 10:**
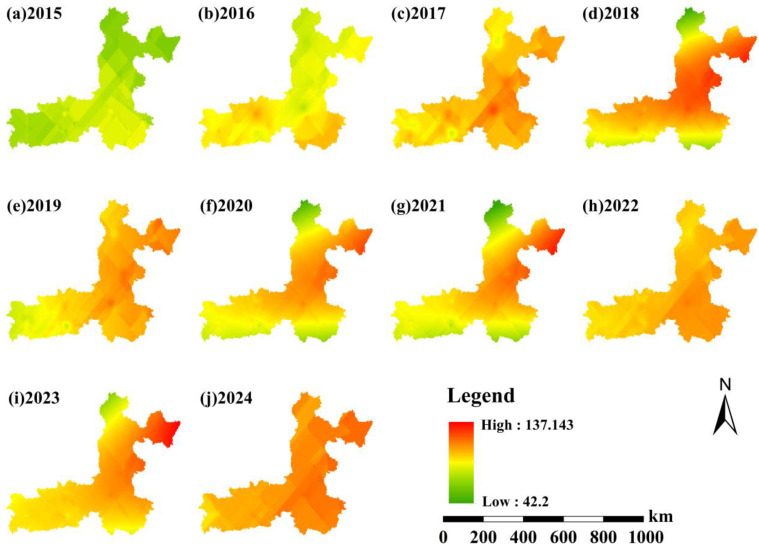
Spatial distribution of annual mean MDA8 O_3_ concentrations in the Fenwei Plain (2015–2024).

The spatial distribution of annual mean MDA8 O_3_ concentrations in the Fenwei Plain exhibits a distinct gradient pattern, characterized by higher levels in the south and west compared to the north and east, alongside pronounced spatial clustering of high-value zones (Figure 10). As illustrated in Figure 1, regions with elevated O_3_ concentrations ($\geq 100\;\mu g/m^{3}$) are predominantly concentrated in southern cities, including XA, XY, WN, and YC, whereas concentrations remain relatively lower in northern areas such as LL and LF.

This spatial heterogeneity is closely coupled with regional photochemical reaction conditions, precursor emission intensities, and meteorological dispersion factors. Southern cities, marked by dense populations and intensive industrial and vehicular activities, exhibit higher concentrations of VOCs and NOx. Coupled with stronger summer solar radiation, these factors create highly favorable conditions for O_3_ formation. Conversely, northern mountainous regions, characterized by higher vegetation cover and limited anthropogenic emissions, show a substantially lower potential for O_3_ generation.

From 2015 to 2024, the overall MDA8 O_3_ concentrations followed a trend of “initial increase followed by stabilization.” Between 2015 and 2020, high-concentration zones expanded and levels in core urban centers climbed steadily, likely driven by rapid regional economic growth, rising precursor emissions, and unfavorable stagnant meteorological conditions (e.g., high temperatures). From 2020 to 2024, however, the previously rapid growth of O_3_ entered a stabilization phase, with concentrations fluctuating at a high level rather than continuing their steep upward trajectory. This shift suggests that the implementation of the ‘Blue Sky Defense Action Plan’ and targeted O_3_ control measures effectively curbed the sharp increasing momentum, even though an absolute decrease in concentrations has not yet been achieved. Consequently, the expansion of high-value areas slowed down, and the spatial concentration gradient became relatively stable.

#### 3.2.3. Spatial Distribution at a Seasonal Scale

**Figure 11:**
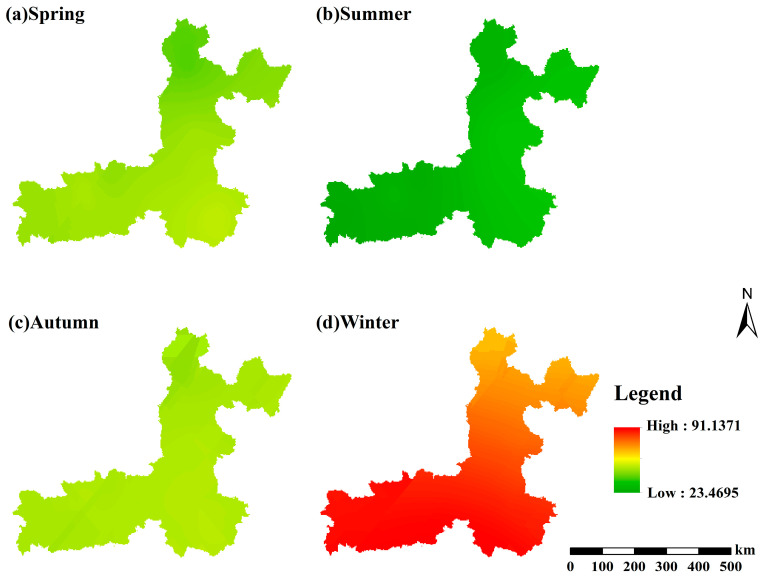
Seasonal spatial distribution of mean PM_2.5_ concentrations in the Fenwei Plain (2015–2024).

From 2015 to 2024, the spatial patterns of seasonal mean PM_2.5_ concentrations in the Fenwei Plain exhibited distinct seasonal variations (Figure 11). In winter, a continuous belt of high-value clusters formed along the Weihe River Valley, with both pollution intensity and spatial extent reaching their annual peaks. This phenomenon is primarily attributed to the surge in coal combustion emissions during the heating season, coupled with unfavorable dispersion conditions such as stagnant meteorological patterns and temperature inversions, which facilitate the persistent accumulation of pollutants.

Spring and autumn serve as transitional periods; although pollution levels decline, the southern plain area maintains a relatively clustered distribution. These patterns are influenced by residual emissions from the heating season and the accumulation of precursors, respectively. In contrast, summer concentrations decrease significantly across the entire region, leading to a more uniform and cleaner spatial distribution. This improvement is driven by enhanced vertical mixing within the atmospheric boundary layer, increased precipitation scavenging efficiency, and reduced emission intensities. Fundamentally, this seasonal spatial heterogeneity results from the coupled regulation of emission strengths and meteorological conditions across various time scales, with heavy winter pollution episodes contributing most significantly to the annual regional mean pollution levels.

**Figure 12:**
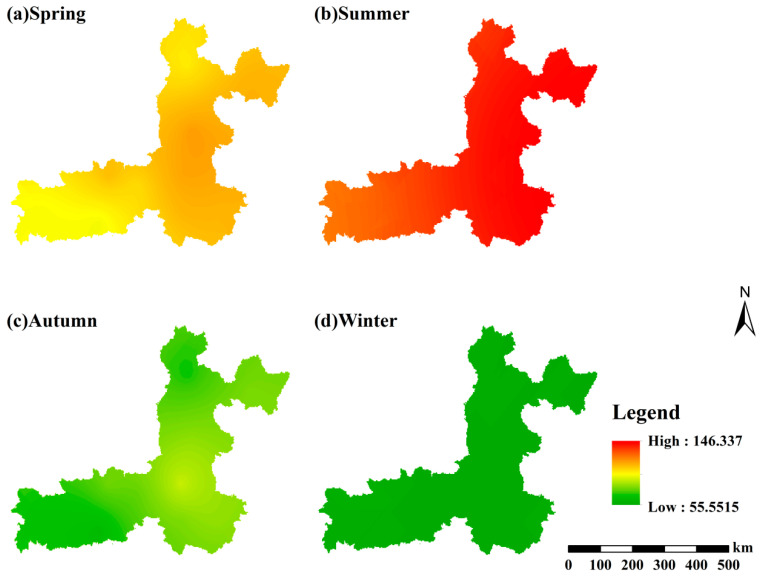
Seasonal spatial distribution of mean MDA8 O_3_ concentrations in the Fenwei Plain (2015–2024).

From 2015 to 2024, the spatial distribution of seasonal mean MDA8 O_3_ concentrations in the Fenwei Plain exhibited a seasonal variation pattern sharply contrasting with that of PM_2.5_ (Figure 12). In summer, a continuous high-value core region emerged in the southern plain, where peak concentrations exceeded $146\;\mu g/m^{3}$. This spatial pattern is closely coupled with intense solar radiation, high temperatures, and elevated precursor emissions (VOCs and NOx). These factors significantly accelerate photochemical reaction rates, driving the persistent formation and accumulation of O_3_ throughout the region.

In spring and autumn, concentration levels were relatively moderate, characterized by a distinct north–south gradient. These distributions were primarily regulated by long-range dust transport in spring and residual precursors in autumn, respectively. Conversely, O_3_ concentrations reached their annual minimum in winter, presenting a low-concentration and relatively uniform spatial distribution. This trend is mainly attributed to weakened solar radiation, atmospheric boundary layer (ABL) compression, and a reduction in precursor emissions. Fundamentally, this seasonal spatial heterogeneity results from the coupled regulation of photochemical reaction intensity, meteorological dispersion conditions, and precursor emissions. Notably, the high-value regions identified in summer represent critical target areas for regional O_3_ pollution prevention and control strategies.

### 3.3. Analysis of PM_2.5_ and O_3_ Compound Pollution

#### 3.3.1. Temporal Characteristics of the Compound Pollution

From the perspective of temporal evolution, the trends in the three categories of pollution days across the Fenwei Plain from 2015 to 2024 exhibit significant divergence. This divergence reflects a profound transition in the regional atmospheric pollution structure, shifting from a traditional “coal-smoke” type to a “photochemical” pollution type.

**Figure 13:**
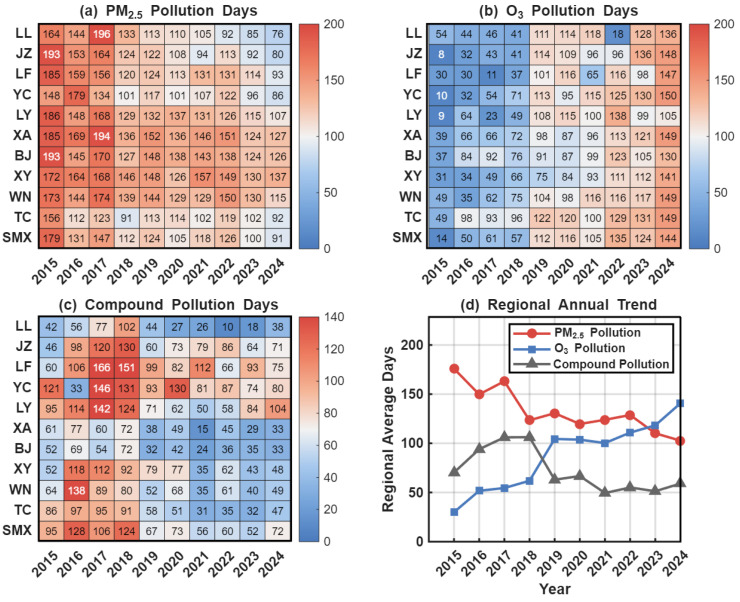
Pollution days and regional trends in the Fenwei Plain from 2015 to 2024. (**a**) PM_2.5_ pollution days; (**b**) MDA8 O_3_ pollution days; (**c**) Compound pollution days; (**d**) Trends in regional average annual pollution days.

PM_2.5_ pollution has been effectively mitigated. As illustrated in Figure 13a,d, the number of PM_2.5_ pollution days generally shows a fluctuating downward trend. The regional average annual pollution days decreased from a peak of over 160 days during 2015–2017 to approximately 100 days in 2022–2024, representing a substantial improvement. This downward trajectory is highly consistent with the rigorous implementation of particulate matter emission reduction measures in the region, such as the “coal-to-gas” conversion and the remediation of “scattered, disordered, and polluting” (SDP) enterprises. These results indicate that traditional particulate matter management has yielded remarkable preliminary success.

In contrast, the O_3_ pollution situation has become increasingly severe. O_3_ pollution days have exhibited a continuous and rapid upward trend. As shown in Figure 13b, O_3_ non-attainment days across all cities were at a relatively low level in 2015 (regional mean < 40 days). However, since 2019, O_3_ pollution days in all cities have surpassed the 100-day threshold, with the regional mean approaching 140 days by 2024. This trend underscores that photochemical pollution has replaced particulate matter as the core bottleneck restricting the continuous improvement of regional air quality.

Compound pollution is characterized by an “initial increase followed by stabilization.” According to Figure 13c, “compound pollution days”—defined as days with simultaneous PM_2.5_ and O_3_ non-attainment—reached a peak in 2017–2018 (regional mean of ~106 days). Although these levels subsequently declined, they remained within the range of 50–60 days in most years. This fluctuation suggests that the rapid surge in O_3_ has partially offset the benefits of PM_2.5_ reduction, maintaining a high risk of concurrent pollutant exceedance.

In summary, while PM_2.5_ management has achieved significant progress, the sharp increase in O_3_ concentrations poses a persistent risk of synergistic non-attainment for both pollutants. Consequently, regional atmospheric pollution control has transitioned from the early stage of single-pollutant management to a new phase involving the synergistic reduction of PM_2.5_ and O_3_, as well as the in-depth remediation of volatile organic compounds (VOCs) and nitrogen oxides (NOx).

**Figure 14:**
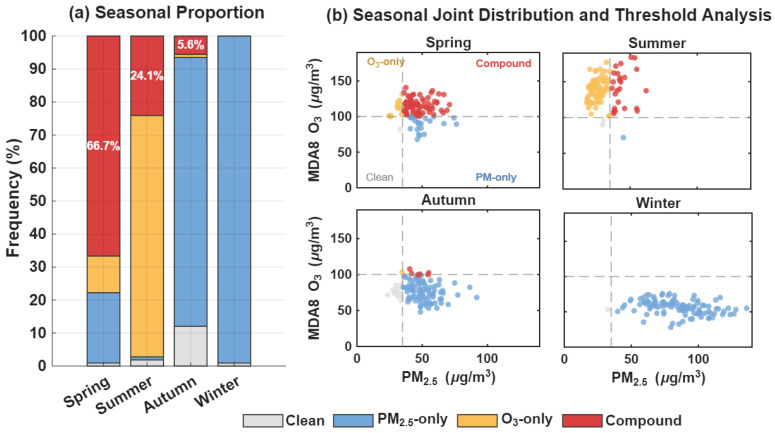
Seasonal characteristics of PM_2.5_ and O_3_ compound pollution in the Fenwei Plain. (**a**) Seasonal proportions of different pollution types. (**b**) Seasonal joint distribution and threshold analysis of PM_2.5_ and MDA8 O_3_ concentrations.

Figure 14 clearly illustrates the distinct seasonal variations of PM_2.5_ and O_3_ compound pollution in the Fenwei Plain. As shown in Figure 14a, the frequency of simultaneous exceedance days for both pollutants peaks in spring (66.7%), followed by a progressive decline in summer (24.1%) and autumn (5.6%). No compound exceedance events were recorded in winter. This underscores that spring represents a critical window for the synergistic prevention and control of PM_2.5_ and O_3_ in the region.

Figure 14b reveals the seasonal successional patterns (i.e., anti-phase relationship) of the two pollutants: PM_2.5_ concentrations peak in winter and reach their minimum in summer, whereas MDA8 O_3_ concentrations exhibit the opposite trend, peaking in summer and reaching their annual low in winter. This distributional discrepancy is primarily attributed to the combined effects of coal-based heating emissions in winter and intensified photochemical reaction mechanisms during summer. Notably, although the absolute concentrations of both pollutants do not reach their annual maximums during spring, the fact that both remain at moderate-to-high levels, coupled with meteorological conditions conducive to their synergistic increase, provides favorable circumstances for the occurrence of compound pollution events.

#### 3.3.2. Spatial Characteristics of Compound Pollution

**Figure 15:**
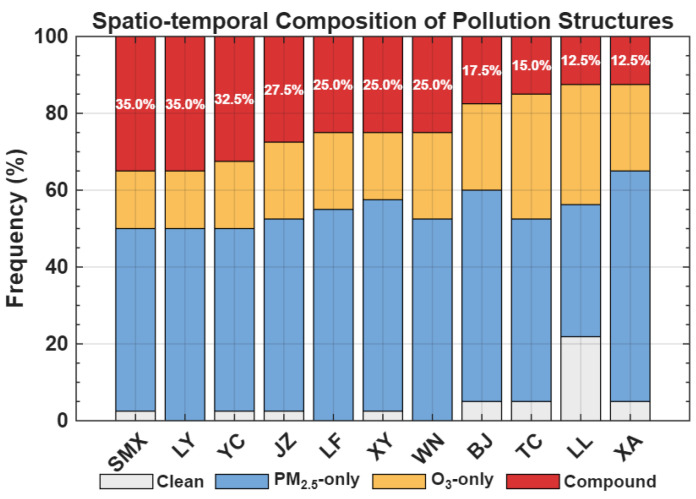
Composition of pollution structures by city across the Fenwei Plain. The red portions represent the compound pollution exceedance ratios.

As illustrated in Figure 15, the pollution structures vary significantly across the cities in the Fenwei Plain. Among them, SMX and LY exhibited the highest compound pollution exceedance ratios (35.0%), followed by YC (32.5%) and JZ (27.5%). In contrast, cities like XA and LL showed relatively lower compound pollution frequencies (12.5%). By analyzing the overall structure, it is evident that PM2.5-only pollution remains a dominant contributor in most cities, while the rising proportion of O3-only and compound pollution underscores the increasing complexity of air quality management in the region.

**Figure 16:**
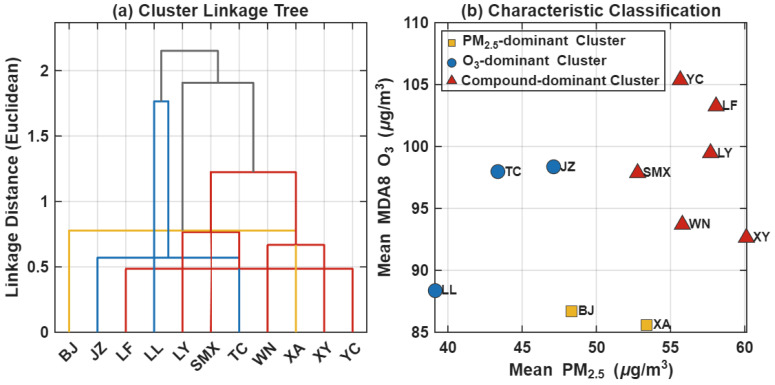
Cluster analysis of PM_2.5_ and O_3_ compound pollution across cities in the Fenwei Plain. (**a**) Hierarchical clustering dendrogram based on Euclidean distance; (**b**) Characteristic classification based on mean PM_2.5_ and MDA8 O_3_ concentrations.

The cluster analysis further elucidates the spatial differentiation patterns of compound pollution across the Fenwei Plain. As illustrated in the hierarchical clustering dendrogram (Figure 16a), the 11 regional cities are categorized into three distinct clusters based on pollution levels and structural discrepancies, exhibiting a strong correlation with their respective geographical locations.

Combined with the characteristic classification plot (Figure 16b), the specific features of each cluster are defined as follows:

(1) Cluster 1 (O_3_-dominant type, e.g., LL, TC, and JZ): Characterized by relatively low PM_2.5_ levels but moderate-to-high MDA8 O_3_ concentrations, with O_3_ serving as the primary pollutant contributor.
(2) Cluster 2 (High compound risk type, e.g., SMX, LY, YC, LF, XY, and WN): These cities exhibit elevated concentrations of both PM_2.5_ and O_3_, representing typical “dual-high” compound pollution features. This cluster serves as the high-pressure zone for regional synergistic prevention and control.
(3) Cluster 3 (PM_2.5_-dominant type, e.g., XA and BJ): O_3_ concentrations are relatively low, with pollution pressure predominantly concentrated in PM_2.5_.

The clustering results demonstrate that compound pollution in the Fenwei Plain is non-homogeneously distributed. Instead, it follows a spatial model characterized by “contiguous high-risk distribution and localized characteristic differentiation.” These findings provide a robust scientific foundation for implementing tailored “one city, one policy” strategies and enhancing regional collaborative governance.

### 3.4. Spatiotemporal Coupling Analysis

#### 3.4.1. Spatiotemporal Evolution Patterns

**Figure 17:**
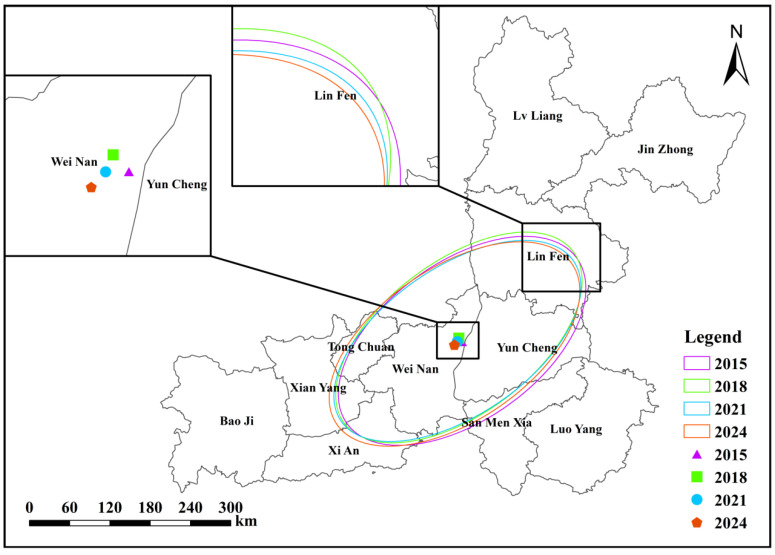
Spatiotemporal evolution of PM_2.5_ concentrations in the Fenwei Plain from 2015 to 2024. (Note: The map illustrates the Standard Deviational Ellipses (SDE) and spatial centroid shifts for the selected years).

During the study period (2015–2024), the spatial distribution of PM_2.5_ concentrations in the Fenwei Plain exhibited significant spatiotemporal evolution characteristics (Figure 17). The Standard Deviational Ellipses (SDE) consistently maintained a Northeast–Southwest (NE–SW) orientation, with the major axis aligning closely with regional topography and prevailing wind directions. The spatial centroid of PM_2.5_ showed a slight migration toward the southwest, remaining primarily within Weinan City and its surrounding areas; this shift was particularly pronounced between 2021 and 2024. The azimuth angle followed a decreasing-then-increasing trend, reaching a minimum of approximately $28.14^{\circ }$ in 2019 before a gradual rebound, reflecting a clockwise adjustment of the distribution axis in recent years. Furthermore, the lengths of both the major and minor axes, as well as the ellipse area, showed an overall shrinking trend, with the area decreasing from $59,906\;km^{2}$ in 2015 to $58,834\;km^{2}$ in 2024 (Table 4). This spatial contraction indicates that PM_2.5_ pollution has become more concentrated, reflecting the preliminary efficacy of regional emission reduction measures in narrowing the scope of high-concentration hotspots.

**Figure 18:**
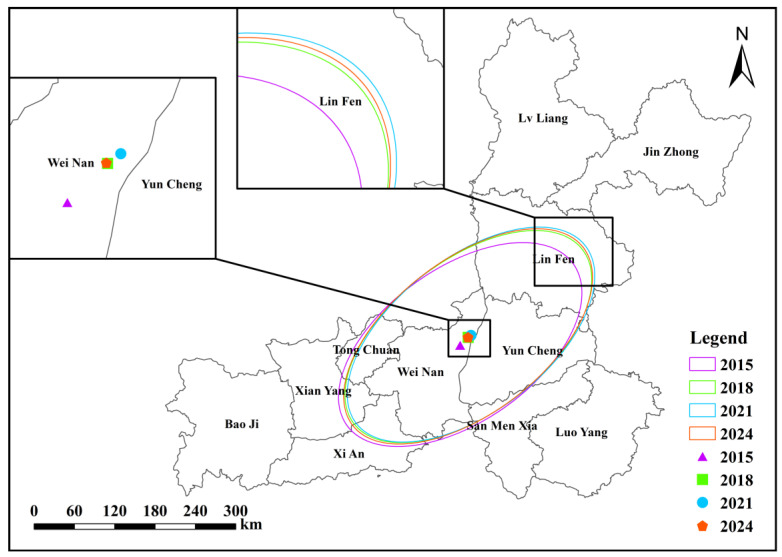
Spatiotemporal evolution of O_3_ concentrations in the Fenwei Plain from 2015 to 2024. (Note: The map illustrates the Standard Deviational Ellipses (SDE) and spatial centroid shifts for the selected years).

From 2015 to 2024, the spatial distribution of annual mean O_3_ concentrations in the Fenwei Plain exhibited significant spatiotemporal dynamics (Figure 18). The Standard Deviational Ellipses (SDE) consistently maintained a Northeast–Southwest (NE–SW) orientation, which aligns closely with the regional prevailing summer winds and the emission patterns of precursors (e.g., VOCs and NOx). This consistency highlights the profound influence of topography and meteorological factors on the spatial configuration of O_3_. During the study period, the spatial centroid of O_3_ pollution shifted slightly toward the northeast, remaining localized near the boundary between WN and YC. Notably, this migration trend was more pronounced between 2015 and 2018, indicating an expansion of high-concentration centers toward southern Shanxi (e.g., YC and LF). The azimuth angle displayed a phased characteristic of “increasing then decreasing,” peaking at approximately $28.92^{\circ }$ in 2019 before a subsequent decline. This variation reflects a subtle adjustment of the O_3_ pollution axis after 2019, suggesting a relative increase in the pollution growth potential or emission contributions from cities in the southwest. Furthermore, the major and minor axes followed a trend of “expansion followed by contraction,” with the total area changing from approximately $60,090\;km^{2}$ in 2015 to $62,804\;km^{2}$ in 2024 (Table 5). This “dispersion followed by concentration” characteristic suggests that O_3_ pollution underwent a broad expansion phase, while recent control measures may have effectively driven the pollution range to concentrate toward core high-value zones.

#### 3.4.2. Spatio-Temporal Cross-Correlation Function (STCCF) Results

**Figure 19:**
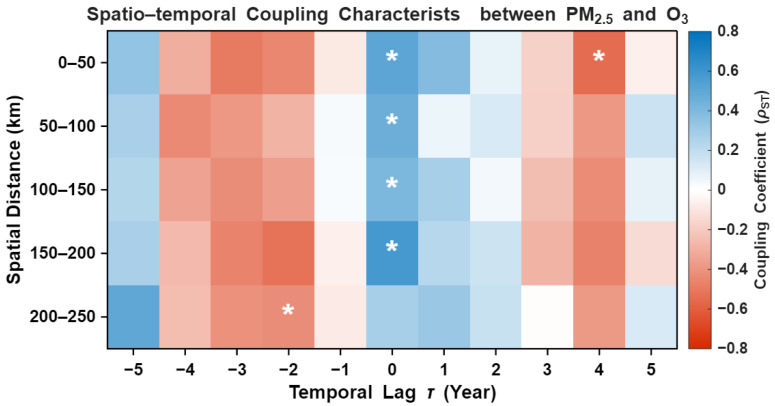
Heatmap of spatiotemporal coupling coefficients between PM_2.5_ and O_3_ in the Fenwei Plain. The asterisk (*) denotes statistically significant correlations (*p* < 0.05).

The results of the Spatio-temporal Cross-Correlation Function (STCCF), derived from detrended and normalized annual series, reveal the intrinsic coupling mechanisms between PM_2.5_ and O_3_ in the Fenwei Plain (Figure 19). By removing the long-term deterministic trends driven by consistent emission reduction policies, the current STCCF specifically captures the synchronized stochastic fluctuations and interannual oscillation characteristics of the two pollutants.

At a temporal lag of τ=0, a significant and robust positive coupling (ρ>0.4, p<0.05) is consistently observed across almost all spatial distance bins (0–200 km). This ubiquitous synchronization, even after detrending, suggests that PM_2.5_ and O_3_ are governed by shared regional driving forces. These are likely attributed to the high sensitivity of their precursor interactions (e.g., the VOCs-NOx synergetic cycle) to interannual meteorological variability, such as the persistence of stagnant atmospheric conditions or regional-scale climate oscillations.

Notably, at a spatial scale of 0–50 km, the coupling strength exhibits a secondary peak at a positive lag of 4 years, whereas a localized significant negative correlation (the “seesaw” effect) is identified at a temporal lag of −2 years for the 200–250 km distance interval. The transition from positive synchronization at τ=0 to scattered lag effects underscores the scale-dependent nature of the PM_2.5_-O_3_ interaction. The dominance of short-term positive synchronization implies that synergistic control strategies will yield immediate co-benefits across the entire Fenwei Plain.

Conclusion and Policy Implications: The identification of a dominant positive coupling on an interannual scale, independent of long-term policy-driven trends, provides a rigorous scientific foundation for the synergistic management of PM_2.5_ and O_3_. Policymakers should transition from trend-based observations to “fluctuation-aware” collaborative governance. Given the high spatial synchronization at Lag 0, cross-regional emergency response measures during meteorologically stagnant years are critical. Strengthening joint emission reductions for shared precursors (e.g., NOx) will effectively leverage this intrinsic positive coupling, leading to a more resilient and synchronized decline in both pollutants across the basin.

#### 3.4.3. Results of the Geographically and Temporally Weighted Regression (GTWR) Model

**Figure 20:**
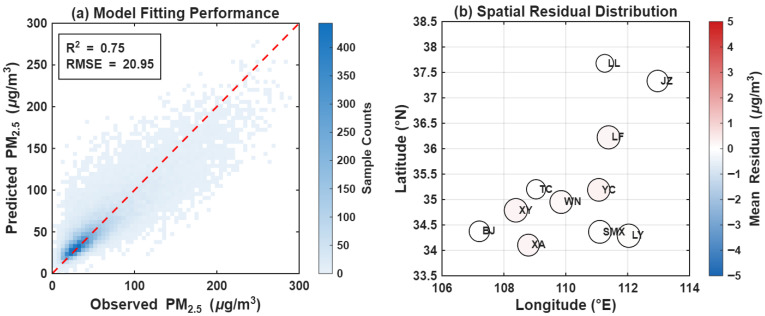
Fitting performance and spatial residual distribution of the PM_2.5_ GTWR model. (**a**) Scatter density plot comparing observed and predicted PM_2.5_ concentrations. (**b**) Spatial distribution of the Mean Residuals across the 11 studied cities.

To validate the effectiveness of the GTWR model in estimating PM_2.5_ concentrations across the Fenwei Plain, we analyzed the model’s fitting performance and residual distribution (Figure 20). The scatter density plot (Figure 20a) reveals that the predicted PM_2.5_ values are highly concentrated along the 1:1 dashed line with the observed values, demonstrating an excellent linear correlation. The fitting results yielded a coefficient of determination ($R^{2}$) of 0.75, indicating that the GTWR model can explain 75% of the spatiotemporal variations in PM_2.5_ within the region. The Root Mean Square Error (RMSE) was calculated at $20.95\;\mu g/m^{3}$. Although a slight underestimation is observed in the extremely high-concentration range, the overall fitting accuracy remains high and is sufficient to support subsequent spatial evolution analysis.

The spatial residual distribution map (Figure 20b) illustrates the prediction bias across different geographical locations. Results show that the Mean Residuals for the 11 cities in the Fenwei Plain remain at a consistently low level, with most circle colors near white (approaching zero). This suggests that the error distribution is relatively uniform across the spatial scale, with no significant systematic biases, such as pronounced regional overestimation or underestimation. Prediction accuracy for core cities, including XA, XY, and WN, is notably stable. In summary, by incorporating geographical locations and temporal lag effects, the GTWR model demonstrates robust mapping capabilities for PM_2.5_ concentrations in the Fenwei Plain, providing scientifically sound and reliable prediction results.

**Figure 21:**
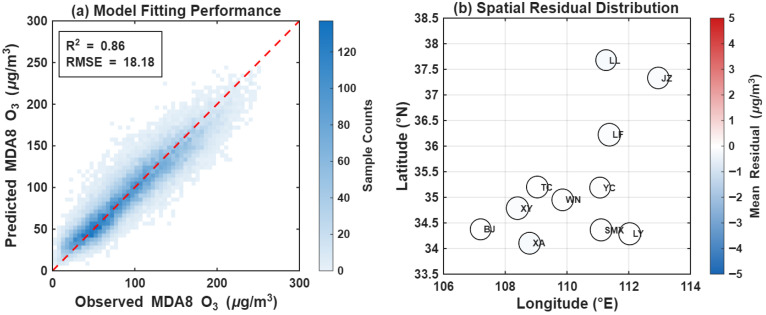
Fitting performance and spatial residual distribution of the MDA8 O_3_ GTWR model. (**a**) Scatter density plot comparing observed and predicted MDA8 O_3_ concentrations; (**b**) Spatial distribution of the Mean Residuals across the 11 studied cities.

To verify the robustness of the MDA8 O_3_ estimation model, we conducted a comprehensive comparison between the observed and predicted data (Figure 21). As illustrated in Figure 21a, the predicted values are evenly distributed along both sides of the 1:1 dashed line, demonstrating exceptionally high consistency with the observed data. Statistical indicators reveal that the overall coefficient of determination ($R^{2}$) reaches 0.86, with a root mean square error (RMSE) of $18.18\;\mu g/m^{3}$. The scatter density is primarily concentrated within the $50-150\;\mu g/m^{3}$ range, indicating that the model is particularly precise in estimating low-to-medium O_3_ concentrations. Although a minor underestimation is observed at extremely high concentrations ($>200\;\mu g/m^{3}$), the overall $R^{2}$ remains at the forefront of similar studies, proving that the model can effectively capture the interannual and seasonal fluctuations of O_3_.

The spatial residual distribution (Figure 21b) further illustrates the prediction bias across various geographical nodes. The results indicate that the Mean Residuals for the 11 major cities (e.g., XA, LY, LF, and LL) consistently fall within the range of [−1, 1], with most circle colors appearing neutral white. This suggests that the model is free from significant spatial systematic biases (Spatial Bias). Whether in topographically complex regions such as LL and JZ, or in the Weihe Valley like XA and XY, the model maintains stable and reliable predictive performance. In summary, the high $R^{2}$ value, coupled with a well-balanced spatial residual distribution, underscores the superior performance of the model in resolving the complex spatiotemporal dynamics of MDA8 O_3_ in the Fenwei Plain. Consequently, the estimation results provide a robust and reliable data source for subsequent investigations into the evolutionary characteristics of regional photochemical pollution.

## 4. Discussion

### 4.1. Evolution of PM_2.5_ and O_3_ Trends and the “See-Saw” Effect

This study demonstrates that between 2015 and 2024, PM_2.5_ concentrations in the Fenwei Plain (FWP) decreased significantly by approximately 18% (from ~50 to 41 μg/m^3^), while MDA8 O_3_ exhibited a fluctuating upward trend, increasing by roughly 47% (from ~75 to 110 μg/m^3^). These results confirm that on a decadal scale, the FWP has entered a deep transitional phase of “PM improvement and O_3_ rebound”. The continuous improvement in PM_2.5_ is primarily attributable to the effective implementation of emission reduction measures such as “coal-to-gas” conversion, the remediation of “scattered, small, and poorly managed” enterprises, and the elimination of backward production capacity during the “Blue Sky Defense War” [47,48,49].

However, the rise in O_3_ may stem from two distinct mechanisms. First, the reduction in PM_2.5_ concentrations enhances surface solar radiation by reducing light scattering and absorption, thereby accelerating photochemical reaction rates. Second, as suggested by the photochemical theories of Schwartz et al., the non-linear relationship between VOCs and NOx emission ratios may push the atmosphere into a regime more sensitive to O_3_ formation following a substantial reduction in fine particulates [50,51,52]. The significant negative correlation identified by the STCCF method at the 150–200 km scale (*p* < 0.05) quantitatively characterizes this synergistic “see-saw” evolution.

### 4.2. Seasonal Characteristics and the Critical Spring Window

The study indicates that compound pollution days in the FWP followed a “increase then stabilize” pattern, with spring identified as the critical window for simultaneous exceedances of both pollutants, accounting for 66.7% of all compound pollution days. This finding holds significant early-warning value. Unlike the pure O_3_ pollution typical of summer, the spring window is characterized by rising temperatures and enhanced solar radiation coupled with frequent dust transport from northern regions [53]. This creates a unique scenario where high background PM_2.5_ overlaps with rapid O_3_ generation.

Furthermore, K-means clustering identified high-risk composite pollution clusters represented by SMX and LY, where the compound exceedance ratio reached 35.0%. This reflects the synergistic effect of high precursor emission intensity in the industrial bases of western Henan and southern Shanxi combined with topographic obstruction. In contrast, while the core Guanzhong area (e.g., XA) faces considerable PM_2.5_ pressure, its compound pollution ratio remains relatively controlled, potentially due to stricter mobile source regulations and more favorable dispersion conditions in recent years.

### 4.3. Spatiotemporal Heterogeneity of Driving Mechanisms and Basin Topography

The GTWR model results reveal the non-stationary driving characteristics of meteorological factors on pollutant distribution. As summarized in Table 6, the regression coefficients vary across space and time, highlighting the localized effects of each driver. The significant negative correlation between temperature and PM_2.5_ is most prominent in certain parts of the Guanzhong Plain. However, on a regional scale, the impact of temperature exhibits notable heterogeneity, reflecting the complex balance between thermal convection and precursor transformation. Crucially, relative humidity (RH) exhibits a predominantly positive correlation across the entire region, with a mean coefficient of 0.432 and positive values in 75% of the samples.

These findings support the perspective of Kumar et al. regarding the localized effects of meteorological factors [54,55].

The unique “mountain-enclosed” basin structure of the FWP acts as an “accelerator” in these driving mechanisms. The physical barriers formed by the Lyuliang Mountains and the northern foot of the Qinling Mountains frequently induce stagnant weather and deep inversion layers during autumn and winter, leading to rapid PM_2.5_ accumulation. In summer, the thermal circulation is constrained by the topography, causing precursors to circulate within the basin and intensifying the potential for secondary O_3_ formation. This explains why the Standard Deviation Ellipse (SDE) consistently maintains a “Northeast–Southwest” orientation, aligning closely with the basin’s terrain and dominant wind directions.

### 4.4. Policy Implications and Limitations

Atmospheric governance in the Fenwei Plain should transition from “single-pollutant control” to “precision-partitioned, synergistic control”. First, given the high incidence of compound pollution in spring, intervention for VOCs and NOx emissions should be advanced. Second, the mid-scale transmission characteristics (150–200 km) necessitate strengthened cross-regional joint prevention and control among cities such as XA, LY, and YC.

While this study analyzes long-term spatiotemporal patterns, limitations remain. Socio-economic indicators (e.g., GDP density, industrial structure) were not integrated into the GTWR analysis. Future research should incorporate human activity data to construct a more comprehensive driving index system. Additionally, the sensitivity of O_3_ increases to precursors (VOC-limited vs. NOx-limited) requires further in-depth verification through numerical modeling at the micro-scale.

## 5. Conclusions

### 5.1. Summary of Findings

This study comprehensively analyzed the decadal spatiotemporal evolution and coupling mechanisms of PM_2.5_ and O_3_ in the Fenwei Plain from 2015 to 2024. The primary findings are as follows:

(1) Pollution Transition: The FWP has transitioned from a particulate-dominated regime to a complex composite pollution phase. While PM_2.5_ concentrations decreased significantly by approximately 32% (from ~60 to 41 $\mu g/m^{3}$), MDA8 O_3_ concentrations rose by 47% (from ~75 to 110 $\mu g/m^{3}$). Spring was identified as the critical window for compound pollution, accounting for 66.7% of simultaneous exceedance events.
(2) Spatial Heterogeneity: Both pollutants exhibited a distinct “Northeast–Southwest” orientation, consistent with the basin’s topography and prevailing wind directions. High-value PM_2.5_ clusters remained along the Weihe River Valley, while O_3_ exhibited a “high in the south, low in the north” gradient. Cluster analysis categorized the 11 cities into O_3_-dominant (e.g., LL, TC), compound high-risk (e.g., SMX, LY, YC, LF), and PM_2.5_-dominant (e.g., XA, BJ) clusters.
(3) Coupling and Drivers: A dominant positive synergy was observed between PM_2.5_ and O_3_ on an annual scale, though a localized “see-saw” effect occurred at specific scales (150–200 km distance with a 3-year lag). The GTWR model demonstrated high robustness in explaining these patterns, achieving $R^{2}$ values of 0.75 and 0.86 for PM_2.5_ and O_3_, respectively.

### 5.2. Policy Implications

The results underscore the necessity of shifting from single-pollutant control to a localized, synergistic governance paradigm.

(1) Synergistic Mitigation: Policymakers should prioritize the synchronized reduction of NOx and VOCs during the spring window to curb the rising trend of O_3_ while maintaining PM_2.5_ improvements.
(2) Regional Cooperation: Strengthening joint prevention and control within the “SMX-LY-YC-LF” industrial corridor is essential to mitigate the high risks of compound pollution observed in the eastern FWP.

### 5.3. Limitations and Future Research

Despite the systematic insights provided, this study has limitations that warrant further investigation.

(1) Statistical vs. Causal Inference: The GTWR model employed in this research is fundamentally a regression-based statistical method. While it effectively captures spatiotemporal heterogeneity and quantifies the strength of associations between pollutants and driving factors, it does not provide direct causal inference. The identified driving mechanisms should be interpreted as statistical correlations rather than definitive causal pathways. Future research could incorporate structural equation modeling (SEM) or causal discovery algorithms to further validate these underlying relationships.
(2) Indicator Integration: The GTWR model primarily focused on meteorological and topographic factors. Future research should integrate socio-economic indicators, such as GDP density and industrial output, to build a more comprehensive driver index.
(3) Micro-scale Mechanisms: While the study quantified spatiotemporal coupling, micro-level photochemical sensitivity (e.g., VOC-limited vs. NOx-limited regimes) remains to be verified through advanced chemical transport modeling or smog chamber experiments.

## Figures and Tables

**Table 1:** Diagnostic metrics for Kriging interpolation performance and their evaluation criteria.

Parameter (Abbreviation)	Mathematical Formula	Optimal Value	Description/Physical Meaning
Mean Error (ME)	$\frac{1}{n}\sum_{i=1}^{n}(Z_{i}-{\hat{Z}}_{i})$	Near 0	Evaluates systematic bias (unbiasedness).
Root Mean Square Error (RMSE)	$\sqrt{\frac{1}{n}\sum_{i=1}^{n}(Z_{i}-{\hat{Z}}_{i}{)}^{2}}$	Minimum	Reflects the overall prediction precision.
Average Standard Error (ASE)	$\sqrt{\frac{1}{n}\sum_{i=1}^{n}\sigma_{i}^{2}}$	Close to RMSE	Measures the uncertainty of predictions.
Mean Standardized Error (MSE)	$\frac{1}{n}\sum_{i=1}^{n}\frac{Z_{i}-{\hat{Z}}_{i}}{\sigma_{i}}$	Near 0	Standardized measure of prediction bias.
Root Mean Square Standardized Error (RMSSE)	$\sqrt{\frac{1}{n}{\sum_{i=1}^{n}\left(\frac{Z_{i}-{\hat{Z}}_{i}}{\sigma_{i}}\right)}^{2}}$	Near 1	Assesses the validity of prediction variability.

**Table 2:** Mann–Kendall (MK) trend test results.

City	PM_2.5_–*Z*	PM_2.5_–*p*	O_3_–*Z*	O_3_–*p*
SMX	−3.76	0.0002	1.97	0.0491
LF	−2.86	0.0042	1.25	0.2105
LL	−2.40	0.0165	1.36	0.1753
XY	−2.33	0.0200	1.79	0.0736
BJ	−3.04	0.0024	1.61	0.1074
JZ	−3.04	0.0024	3.22	0.0013
LY	−2.50	0.0123	1.43	0.1524
WN	−2.68	0.0073	0.72	0.4743
XA	−2.50	0.0123	2.15	0.0318
YC	−3.22	0.0013	0.36	0.7205
TC	−3.22	0.0013	−0.54	0.5915
FWP	−3.04	0.0024	1.79	0.0736

**Table 3:** Cross-validation and structural parameters of different semivariogram models for PM_2.5_ and MDA8 O_3_.

Pollutant	Semivariogram	ME	RMSE	MSE	RMSSE	ASE
PM2.5	Circular function	0.245674	10.869	0.024234	1.00459	10.835
	Gaussian function	0.340854	10.858	0.031480	1.014292	10.717
	Spherical function	0.306587	10.860	0.028864	1.010732	10.758
	Exponential function	0.203603	10.547	0.025674	0.970326	10.910
MDA8 O3	Circular function	0.089566	12.350	0.008338	0.976110	12.649
	Gaussian function	0.169454	12.553	0.014044	1.010758	12.620
	Spherical function	0.117171	12.157	0.010147	0.965956	12.478
	Exponential function	−0.024892	12.189	0.001127	0.937975	12.804

**Table 4:** Standard deviational ellipse (SDE) parameters for PM_2.5_.

Year	Centroid Longitude (°E)	Centroid Latitude (°N)	Major Axis (km)	Minor Axis (km)	Azimuth (°)	Area (km^2^)
2015	110.392	35.245	402.89	189.32	28.69	59,905.66
2016	110.333	35.198	404.36	179.80	28.98	57,101.16
2017	110.364	35.258	404.79	185.23	30.46	58,889.80
2018	110.350	35.257	403.95	183.89	30.54	58,339.71
2019	110.303	35.178	399.10	179.95	28.14	56,405.93
2020	110.295	35.196	401.70	179.60	28.86	56,663.22
2021	110.330	35.217	401.89	177.05	29.01	55,883.27
2022	110.262	35.161	407.98	173.20	28.53	55,496.89
2023	110.276	35.170	403.01	176.43	28.29	55,843.24
2024	110.296	35.192	406.39	184.33	28.31	58,833.47

**Table 5:** Standard deviational ellipse (SDE) parameters for O_3_.

Year	Centroid Longitude (°E)	Centroid Latitude (°N)	Major Axis (km)	Minor Axis (km)	Azimuth (°)	Area (km^2^)
2015	110.247	35.242	391.11	195.62	26.37	60,090.99
2016	110.224	35.233	401.87	197.69	25.91	62,397.20
2017	110.352	35.312	401.33	194.32	27.49	61,252.86
2018	110.355	35.328	401.62	196.139	28.15	61,869.16
2019	110.398	35.358	402.07	197.107	28.92	62,243.25
2020	110.365	35.342	404.68	195.98	28.61	62,288.51
2021	110.391	35.349	404.41	196.97	28.68	62,562.40
2022	110.339	35.301	404.56	195.81	27.86	62,217.13
2023	110.370	35.341	410.06	196.73	28.64	63,359.66
2024	110.352	35.330	406.50	196.71	28.63	62,803.71

**Table 6:** Statistical summary of the spatiotemporal regression coefficients derived from the GTWR model for the Fenwei Plain (2015–2024).

Predictors	Mean	Std.Dev.	Min
RH	0.432	0.691	−1.743
ME	0.244	1.924	−12.357
MW	0.884	1.930	−10.848
PRE	−2.603	5.310	−68.172
SD	−1.103	2.097	−11.973
DEM	0.059	0.096	−0.533

## Data Availability

The original contributions presented in this study are included in the article. Further inquiries can be directed to the corresponding author.

## References

[1] Feng S., Gao D., Liao F., Zhou F., Wang X. (2016). The Health Effects of Ambient PM_2.5_ and Potential Mechanisms. Ecotoxicol. Environ. Saf..

[2] Thangavel P., Park D., Lee Y.-C. (2022). Recent Insights into Particulate Matter (PM_2.5_)-Mediated Toxicity in Humans: An Overview. Int. J. Environ. Res. Public Health.

[3] Zhu Y., Xie J., Huang F., Cao L. (2020). Association between Short-Term Exposure to Air Pollution and COVID-19 Infection: Evidence from China. Sci. Total Environ..

[4] Basith S., Manavalan B., Shin T.H., Park C.B., Lee W.-S., Kim J., Lee G. (2022). The Impact of Fine Particulate Matter 2.5 on the Cardiovascular System: A Review of the Invisible Killer. Nanomaterials.

[5] Xiao Q., Geng G., Xue T., Liu S., Cai C., He K., Zhang Q. (2022). Tracking PM_2.5_ and O_3_ Pollution and the Related Health Burden in China 2013–2020. Environ. Sci. Technol..

[6] Cao J.J., Cui L. (2021). Current Status, Characteristics and Causes of Particulate Air Pollution in the Fenwei Plain, China: A Review. J. Geophys. Res. Atmos..

[7] Peng S., Ju T., Liang Z., Li M., Liu S., Pan B. (2022). Analysis of Atmospheric Ozone in Fenwei Plain Based on Remote Sensing Monitoring. Environ. Monit. Assess..

[8] Feng Y., Ning M., Lei Y., Sun Y., Liu W., Wang J. (2019). Defending Blue Sky in China: Effectiveness of the “Air Pollution Prevention and Control Action Plan” on Air Quality Improvements from 2013 to 2017. J. Environ. Manag..

[9] Jiang X., Li G., Fu W. (2021). Government Environmental Governance, Structural Adjustment and Air Quality: A Quasi-Natural Experiment Based on the Three-Year Action Plan to Win the Blue Sky Defense War. J. Environ. Manag..

[10] Li K., Jacob D.J., Liao H., Shen L., Zhang Q., Bates K.H. (2019). Anthropogenic Drivers of 2013–2017 Trends in Summer Surface Ozone in China. Proc. Natl. Acad. Sci. USA.

[11] Yang Z., Yang J., Li M., Chen J., Ou C.-Q. (2021). Nonlinear and Lagged Meteorological Effects on Daily Levels of Ambient PM_2.5_ and O_3_: Evidence from 284 Chinese Cities. J. Clean. Prod..

[12] Liu X., Yi G., Zhou X., Zhang T., Bie X., Li J., Tan H. (2023). Spatio-Temporal Variations of PM_2.5_ and O_3_ in China during 2013–2021: Impact Factor Analysis. Environ. Pollut..

[13] Mirzaei M., Amanollahi J., Tzanis C.G. (2019). Evaluation of Linear, Nonlinear, and Hybrid Models for Predicting PM_2.5_ Based on a GTWR Model and MODIS AOD Data. Air Qual. Atmos. Health.

[14] Yang Z., Ren Y., Shen L., Liao X., Kwan M.-P. (2025). Spatiotemporal Evolutions and Drivers of Ground-Level Ozone in China (2015–2020): A GTWR-Kriging Approach. Environ. Res..

[15] Li Y., Wang X., Li J., Zhu L., Chen Y. (2022). Numerical Simulation of Topography Impact on Transport and Source Apportionment on PM_2.5_ in a Polluted City in Fenwei Plain. Atmosphere.

[16] She Y., Chen Q., Ye S., Wang P., Wu B., Zhang S. (2022). Spatial-Temporal Heterogeneity and Driving Factors of PM_2.5_ in China: A Natural and Socioeconomic Perspective. Front. Public Health.

[17] Miao Y., Guo J., Liu S., Liu H., Zhang G., Yan Y., He J. (2017). Relay Transport of Aerosols to Beijing-Tianjin-Hebei Region by Multi-Scale Atmospheric Circulations. Atmos. Environ..

[18] Lyu Y., Wu D., Han F., Zhang H., Lv F., Kang A., Hu Y., Pang X. (2025). Co-Occurring Extremes of PM_2.5_ and Ozone in Warm Seasons of the Yangtze River Delta of China: Insights from Explainable Machine Learning. ACS ES&T Air.

[19] Wu X., Xin J., Zhang W., Gao W., Ma Y., Ma Y., Wen T., Liu Z., Hu B., Wang Y. (2022). Variation Characteristics of Air Combined Pollution in Beijing City. Atmos. Res..

[20] Wu S., Yan X., Yao J., Zhao W. (2023). Quantifying the Scale-Dependent Relationships of PM_2.5_ and O_3_ on Meteorological Factors and Their Influencing Factors in the Beijing-Tianjin-Hebei Region and Surrounding Areas. Environ. Pollut..

[21] Jia C., Yan G., Yu X., Li X., Xue J., Wang Y., Cao Z. (2025). Evidence for Coordinated Control of PM_2.5_ and O_3_: Long-Term Observational Study in a Typical City of Central Plains Urban Agglomeration. Toxics.

[22] Zhang Y.-L., Cao F. (2015). Fine Particulate Matter (PM_2.5_) in China at a City Level. Sci. Rep..

[23] Li K., Jacob D.J., Shen L., Lu X., De Smedt I., Liao H. (2020). Increases in Surface Ozone Pollution in China from 2013 to 2019: Anthropogenic and Meteorological Influences. Atmos. Chem. Phys..

[24] Zhao S., Yin D., Yu Y., Kang S., Qin D., Dong L. (2020). PM_2.5_ and O_3_ Pollution during 2015–2019 over 367 Chinese Cities: Spatiotemporal Variations, Meteorological and Topographical Impacts. Environ. Pollut..

[25] Yin Z., Yuan L., Yang Y., Wu X., Chen Z., Long H. (2025). Exploring the Altitude Differentiation and Influencing Factors of PM2.5 and O3: A Case Study of the Fenwei Plain, China. Front. Environ. Sci..

[26] Xiao C., Jingbo Z., Meng F., Cullen J., Wang X., Zhu Y. (2022). Regional Characteristics and Spatial Correlation of Haze Pollution: Interpretative System Analysis in Cities of Fenwei Plain in China. SSRN Electron. J..

[27] Xu L., Wang B., Wang Y., Zhang H., Xu D., Zhao Y., Zhao K. (2025). Characterization and Source Apportionment Analysis of PM_2.5_ and Ozone Pollution over Fenwei Plain, China: Insights from PM_2.5_ Component and VOC Observations. Toxics.

[28] Zhang H., Zhang C., Liu S., Yin S., Zhang S., Zhu H., Yan F., Yang H., Ru X., Liu X. (2025). Insights into the Source Characterization, Risk Assessment and Ozone Formation Sensitivity of Ambient VOCs at an Urban Site in the Fenwei Plain, China. J. Hazard. Mater..

[29] Jia M., Jiang F., Evangeliou N., Eckhardt S., Huang X., Ding A., Stohl A. (2023). Rapid Decline of Carbon Monoxide Emissions in the Fenwei Plain in China during the Three-Year Action Plan on Defending the Blue Sky. J. Environ. Manag..

[30] Xu W., Wang Y., Sun S., Yao L., Li T., Fu X. (2022). Spatiotemporal Heterogeneity of PM2.5 and Its Driving Difference Comparison Associated with Urbanization in China’s Multiple Urban Agglomerations. Environ. Sci. Pollut. Res..

[31] Shukla K., Kumar P., Mann G.S., Khare M. (2020). Mapping Spatial Distribution of Particulate Matter Using Kriging and Inverse Distance Weighting at Supersites of Megacity Delhi. Sustain. Cities Soc..

[32] Di Q., Amini H., Shi L., Kloog I., Silvern R., Kelly J., Sabath M.B., Choirat C., Koutrakis P., Lyapustin A. (2019). An Ensemble-Based Model of PM2.5 Concentration across the Contiguous United States with High Spatiotemporal Resolution. Environ. Int..

[33] Etherington T.R. (2020). Discrete Natural Neighbour Interpolation with Uncertainty Using Cross-Validation Error-Distance Fields. PeerJ Comput. Sci..

[34] Michaelsen J. (1987). Cross-Validation in Statistical Climate Forecast Models. J. Clim. Appl. Meteorol..

[35] Risk C., James P.M.A. (2022). Optimal Cross-Validation Strategies for Selection of Spatial Interpolation Models for the Canadian Forest Fire Weather Index System. Earth Space Sci..

[36] Nedyalkova M., Simeonov V. (2019). Chemomertic Risk Assessment of Soil Pollution. Open Chem..

[37] Marín Celestino A., Martínez Cruz D., Otazo Sánchez E., Gavi Reyes F., Vásquez Soto D. (2018). Groundwater Quality Assessment: An Improved Approach to K-Means Clustering, Principal Component Analysis and Spatial Analysis: A Case Study. Water.

[38] Tariq S., Mariam A., ul-Haq Z., Mehmood U. (2022). Spatial and Temporal Variations in PM2.5 and Associated Health Risk Assessment in Saudi Arabia Using Remote Sensing. Chemosphere.

[39] Zhao Y., Wu Q., Wei P., Zhao H., Zhang X., Pang C. (2022). Explore the Mitigation Mechanism of Urban Thermal Environment by Integrating Geographic Detector and Standard Deviation Ellipse (SDE). Remote Sens..

[40] Shi S., Li M., Li Z., Xi J. (2023). Spatial Heterogeneity and Influencing Factors of High-Grade Tourist Attractions in the Tibetan Plateau. Int. J. Environ. Res. Public Health.

[41] Wang H., Zhao M., Huang X., Song X., Cai B., Tang R., Sun J., Han Z., Yang J., Liu Y. (2024). Improving Prediction of Soil Heavy Metal(Loid) Concentration by Developing a Combined Co-Kriging and Geographically and Temporally Weighted Regression (GTWR) Model. J. Hazard. Mater..

[42] Liu L., Liu Y., Cheng F., Yu Y., Wang J., Wang C., Nong L., Deng H. (2024). Remote Sensing Estimation of Regional PM_2.5_ Based on GTWR Model -a Case Study of Southwest China. Environ. Pollut..

[43] Wang Q., Li J., Yang J., Chen Y., Li Y., Li S., Xie C., Chen C., Wang L., Wang L. (2020). Seasonal Characterization of Aerosol Composition and Sources in a Polluted City in Central China. Chemosphere.

[44] Li L., Lu C., Chan P.-W., Zhang X., Yang H.-L., Lan Z.-J., Zhang W.-H., Liu Y.-W., Pan L., Zhang L. (2020). Tower Observed Vertical Distribution of PM2.5, O3 and NOx in the Pearl River Delta. Atmos. Environ..

[45] Zhang K., Xiu G., Zhou L., Bian Q., Duan Y., Fei D., Wang D., Fu Q. (2018). Vertical Distribution of Volatile Organic Compounds within the Lower Troposphere in Late Spring of Shanghai. Atmos. Environ..

[46] Nguyen T.P.M., Bui T.H., Nguyen M.K., Nguyen T.H., Vu V.T., Pham H.L. (2022). Impact of COVID-19 Partial Lockdown on PM_2.5_, SO_2_, NO_2_, O_3_, and Trace Elements in PM_2.5_ in Hanoi, Vietnam. Environ. Sci. Pollut. Res..

[47] Cheng J., Su J., Cui T., Li X., Dong X., Sun F., Yang Y., Tong D., Zheng Y., Li Y. (2019). Dominant Role of Emission Reduction in PM_2.5_ Air Quality Improvement in Beijing during 2013–2017: A Model-Based Decomposition Analysis. Atmos. Chem. Phys..

[48] Zhang Q., Geng G. (2019). Impact of Clean Air Action on PM2.5 Pollution in China. Sci. China Earth Sci..

[49] Zhang Q., Zheng Y., Tong D., Shao M., Wang S., Zhang Y., Xu X., Wang J., He H., Liu W. (2019). Drivers of Improved PM_2.5_ Air Quality in China from 2013 to 2017. Proc. Natl. Acad. Sci. USA.

[50] Zhao H., Chen K., Liu Z., Zhang Y., Shao T., Zhang H. (2021). Coordinated Control of PM_2.5_ and O_3_ Is Urgently Needed in China after Implementation of the “Air Pollution Prevention and Control Action Plan”. Chemosphere.

[51] Wang T., Xue L., Brimblecombe P., Lam Y.F., Li L., Zhang L. (2017). Ozone Pollution in China: A Review of Concentrations, Meteorological Influences, Chemical Precursors, and Effects. Sci. Total Environ..

[52] Maji K.J., Ye W.-F., Arora M., Nagendra S.M.S. (2019). Ozone Pollution in Chinese Cities: Assessment of Seasonal Variation, Health Effects and Economic Burden. Environ. Pollut..

[53] Nie Y., Yan Y., Ji Y., Gao R., Ren Y., Bi F., Shang F., Li J., Chu W., Li H. (2025). Assessing the PM2.5–O3 Correlation and Unraveling Their Drivers in Urban Environment: Insights from the Bohai Bay Region, China. Atmosphere.

[54] Dai H., Zhu J., Liao H., Li J., Liang M., Yang Y., Yue X. (2021). Co-Occurrence of Ozone and PM_2.5_ Pollution in the Yangtze River Delta over 2013–2019: Spatiotemporal Distribution and Meteorological Conditions. Atmos. Res..

[55] Ma X., Yin Z., Cao B., Wang H. (2023). Meteorological Influences on Co-Occurrence of O_3_ and PM_2.5_ Pollution and Implication for Emission Reductions in Beijing-Tianjin-Hebei. Sci. China Earth Sci..

